# Computational design of novel chimeric multiepitope vaccine against bacterial and viral disease in tilapia (*Oreochromis* sp.)

**DOI:** 10.1038/s41598-024-64383-z

**Published:** 2024-06-18

**Authors:** Ansaya Pumchan, Porranee Proespraiwong, Orathai Sawatdichaikul, Thararat Phurahong, Ikuo Hirono, Sasimanas Unajak

**Affiliations:** 1 Department of Biochemistry, Faculty of Science, Kasetsart University, 50 Ngam Wong Wan, Chatuchak, 10900 Bangkok Thailand; 2 Kasetsart Vaccines and Bio-Product Innovation Centre, Kasetsart University, 50 Ngam Wong Wan, Chatuchak, 10900 Bangkok Thailand; 3 Department of Nutrition and Health, Institute of Food Research and Product Development, Kasetsart University, 50 Ngam Wong Wan, Chatuchak, 10900 Bangkok Thailand; 4 Graduate School of Marine Science and Technology, Tokyo University of Marine Science and Technology, Konan 4-5-7, Minato-KU, Tokyo 108-8477 Japan

**Keywords:** Chimeric multiepitope vaccine (CMEV), Fish diseases, Structural vaccine design, Bacterial disease, Fish vaccine, Biochemistry, Biotechnology, Computational biology and bioinformatics, Molecular biology

## Abstract

Regarding several infectious diseases in fish, multiple vaccinations are not favorable. The chimeric multiepitope vaccine (CMEV) harboring several antigens for multi-disease prevention would enhance vaccine efficiency in terms of multiple disease prevention. Herein, the immunogens of tilapia’s seven pathogens including *E. tarda*, *F. columnare*, *F. noatunensis*, *S. iniae*, *S. agalactiae*, *A. hydrophila,* and TiLV were used for CMEV design. After shuffling and annotating the B-cell epitopes, 5,040 CMEV primary protein structures were obtained. Secondary and tertiary protein structures were predicted by AlphaFold2 creating 25,200 CMEV. Proper amino acid alignment in the secondary structures was achieved by the Ramachandran plot. In silico determination of physiochemical and other properties including allergenicity, antigenicity, glycosylation, and conformational B-cell epitopes were determined. The selected CMEV (OSLM0467, OSLM2629, and OSLM4294) showed a predicted molecular weight (MW) of 70 kDa, with feasible sites of *N-* and *O-*glycosylation, and a number of potentially conformational B-cell epitope residues. Molecular docking, codon optimization, and *in-silico* cloning were tested to evaluate the possibility of protein expression. Those CMEVs will further elucidate in vitro and in vivo to evaluate the efficacy and specific immune response. This research will highlight the new era of vaccines designed based on in silico structural vaccine design.

## Introduction

To date, aquatic animal food has become the crucial animal source of protein for human consumption globally. Notionally, consumer demand for aquaculture production from approximately 82 million metric tons (MT) in 2018 might reach up to 129 million MT by 2050^1^. The industries of freshwater fish production including tilapia increased dramatically in recent years. Tilapia is one of the most pivotal fish species to supply protein nutrition for the worldwide population and its farming has been distributed over 135 countries^2^. Tilapia can resist various environmental conditions, including low dissolved oxygen and alkaline and osmotic stresses. Unfortunately, decreasing tilapia productivity has been reported due to disease outbreaks, especially in intensive-density farming. Among the disease causative agents, the prevalent and virulent pathogens causing severe tilapia mortality are bacteria and viruses such as *Streptococcus*, *Aeromonas*, *Flavobacterium*, *Edwardsiella*, *Francisella*, and TiLV^3^. For preventing and controlling pathogenic distribution, health management such as good husbandry practice, rapid detection, and immunotherapies should be considered. Most notably, one of the effective and acceptable strategies for disease handling is applying vaccines. Until now, various types of tilapia vaccines have been developed including attenuated, inactivated, recombinant, and chimeric vaccines^4^. Remarkedly, most of the available vaccines were generated as monovalent vaccines^5,6^, while some are bivalent^7,8^. Occasionally, co-infections of multiple pathogens can occur during tilapia culture. This phenomenon significantly impacts fish potency in response to the vaccines^9^. To enhance the disease protective strategies for co-infectious outbreaks, thus, the development of polyvalent formulation should be more focused.

Literately, only a small number of multivalent tilapia vaccines have been reported. For example, a formalin-inactivated vaccine of four combined bacterial species *Streptococcus agalactiae*, *S. iniae*, *Lactococcus garvieae,* and *Enterococcus faecalis* mixed with Montanide adjuvant was tested. It showed immune stimulation in Nile tilapia^10^. However, the formulation of an inactivated polyvalent vaccine by mixing more than four bacterial species or combining different pathogen types is still limited in finfish species including tilapia. Hence, at present, an advanced strategy regarding reverse vaccinology and structural vaccinology approaches has been introduced for generating chimeric multiepitope vaccines against infectious diseases in tilapia.

Previously, several tilapia multiepitope vaccines based on *in-silico* design and immunoinformatic approach were reported against some pathogens such as *Aeromonas veronii*^11^, *Edwardsiella ictaluri*^12^, *S. agalactiae*^13^, and TiLV^14^. Nevertheless, prior currently reported chimeric vaccines were mostly constructed as monovalent and bivalent forms. Therefore, in this study, a new platform of polyvalent chimeric multiepitope vaccine would be created depending on seven tilapia infectious pathogens namely six bacterial species such as *E. tarda*, *Flavobacterium columnare*, *Francisella noatunensis*, *S. iniae*, *S. agalactiae*, and *A. hydrophila* and one virus as TiLV. The predicted conserved B-cell epitopes, identified from immunogens of those pathogenic agents, were joined and shuffled with the specific linker for constructing the chimeric multiepitope vaccine (CMEV). Numerous bioinformatics tools and servers were employed to validate their folding structures, physiochemical characteristics, and other crucial features. Since the recognition between vaccine ligand and immune cell receptor is essential for eliciting the host’s immune response. Herein, a representative of pattern recognition receptors as the toll-like receptor 4 (TLR4), which is responsible as the bacterial sensor^15^, was employed for studying the molecular docking with the constructed CMEV candidates. Lastly, the desirably selected CMEVs were subsequently optimized and synthesized for further study of the expression in both prokaryotic and eukaryotic systems.

## Results

### Immunogen selection and B-cell epitope identification

To generate the chimeric multiepitope vaccine (CMEV), immunogenic proteins from six pathogenic bacteria (*A. hydrophila*, *E. tarda*, *F. columnare*, *F. noatunensis*, *S. iniae*, *S. agalactiae*) and one virus (TiLV) were identified based on representing high possibility to be efficient vaccine candidates from previously published reports (Table 1 and Supplementary Table 1). Notably, most of the selected immunogens provided favorable relative survival percentages (%RPS) ranging from 55.00% to 98.59%. The consensus amino acid sequences were identified among several pathogen species. The antigenic peptides and B-cell epitopes were characterized from all those conserved amino acid sequences using multiple prediction servers to sift only the obtained consensus epitopes among the different framework predictions. Regarding this step, summarily, 117 sequences of predicted epitopes from 34 immunogens^16–33^ were obtained (Supplementary Table 1). Subsequently, the epitopes were screened according to 4 criteria: serving at least 2 epitope prediction servers, containing at least 19 residues, carrying low hydrophobicity, and holding alpha-helix structure. Remarkedly, a total of 21 desired candidates belonging to seven pathogens were chosen (Table 1 and Supplementary Table 2) for designing the CMEV. Notably, the predicted epitopes with long sequences, high hydrophilicity, and flexible structure (with beta-turn and alpha-helix) could be more accessible and recognized by the antibody^34^.

**Table 1:** The selected B-cell epitopes of constructed CMEV candidates.

Pathoge (No. of total epitope)	Gene	Protein name	Epitope sequence	Accession No
*Edwardsiella tarda *(2)	*DegP*	Serine endoprotease	AQALPSLAPMLEKVMPSVVSINVE	PVD94192.1
	*Eta2*	Skp-like molecular chaperone	EKIAVVNVASVFQQLPQRDAVAKQ	ADR31356.1
*Flavobacterium columnare *(3)	*OmeP*	Outer membrane efflux protein precursor	RQLYTSQQAYLQSMVEVITK	ANO47148.1
	*DnaK*	Molecular chaperone DnaK	KFEQLADSLVKRSMEPVVKAL	PDS21884.1
			DIDEVILVGGSTRIPVIQEQVEKF	
*Francisella noatunensis *(4)	*ClpB*	ATP-dependent chaperone ClpB	RKPYSVILLDEVEKAHADIFNILLQVLDD	PLR04593.1
			EIAKIQIKRLEKRLADLSIGLEV	
	*GroEL*	Molecular chaperone GroEL	HGIALLRKAIEAPLRQIVSNAGGESSVVVNQVK	ALK94214.1
	*IglB*	Intracellular growth locus protein B	KYIQKVITVIDKLIDLQVNSII	ABK90199.1
*Streptococcus iniae *(3)	*Sip11*	Periplasmic_Binding_Protein_Type_2	DYDYVIKHKEDIVKHYSDIFVDLQS	ADE61838.1
	*Eno*	Enolase	KYNQLLRIEDQLGEVAQYKGIK	EKB52954.1
	*Gapdh*	NADP-dependent glyceraldehyde-3-phosphate dehydrogenase	KRVLVMDKVADQLIANVKTLVDKLSI	RLU98986.1
Tilapia lake virus: TiLV (3)	*Tis1*	Hypothetical protein	AIFLSHPFFRLLSSVVETHARSVLSKVSAVYTA	QJD15201.1
	*Tis5*	Hypothetical protein	KQVPFYGSIKVLVFRRLRVVCFKTFFY	QAB07939.1
	*Tis6*	Hypothetical protein	HFYLQDCPMSWLRVIRTLTLFSTLFS	AWK60418.1
*Aeromonas hydrophila *(3)	*OmpA*	Porin OmpA	PEGVAALNTLYQQIVDVQPKDGSAVVVGYT	AVP83691.1
	*Tdr*	TonB-dependent receptor	TVYVNGRYHINDSVDFVPQLIGSRVT	ORJ65912.1
	*Aer*	Aerolysin	TVSVEARPTVPPHSSLPVRVALYKSN	KRW48464.1
*Streptococcus agalactiae *(3)	*Bac*	C protein beta antigen	DALLELENQFNETNRLLHIKQ	BAE45252
			KIAVSKYMSKVLDGVHQHLQK	
	*Spb*	Surface protein Spb1	GTEKVYQYVIKDTMPSASVVDL	WP_000913277

The number in the blanket indicates the total number of identified epitopes of each pathogen.

### Chimeric multiepitope vaccine (CMEV) construction and molecular modeling

**Figure 1:**
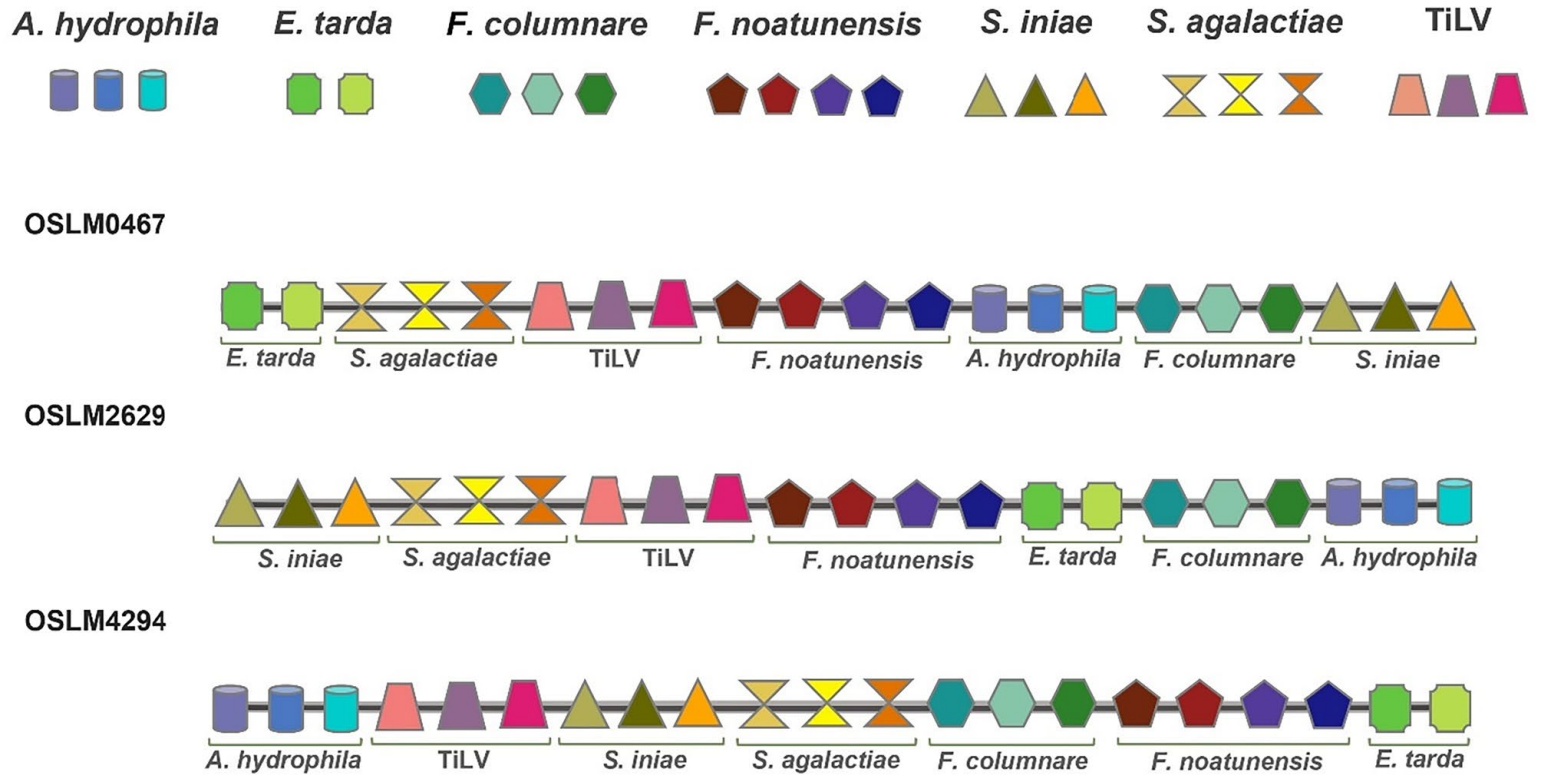
The schematic diagram represented the selected CMEV candidates comprising 21 epitopes from 7 pathogens. Each shape indicated each pathogen, while each color indicated each selected epitope, and the gap in gray color indicated (Gly)_8_ linkers.

**Figure 2:**
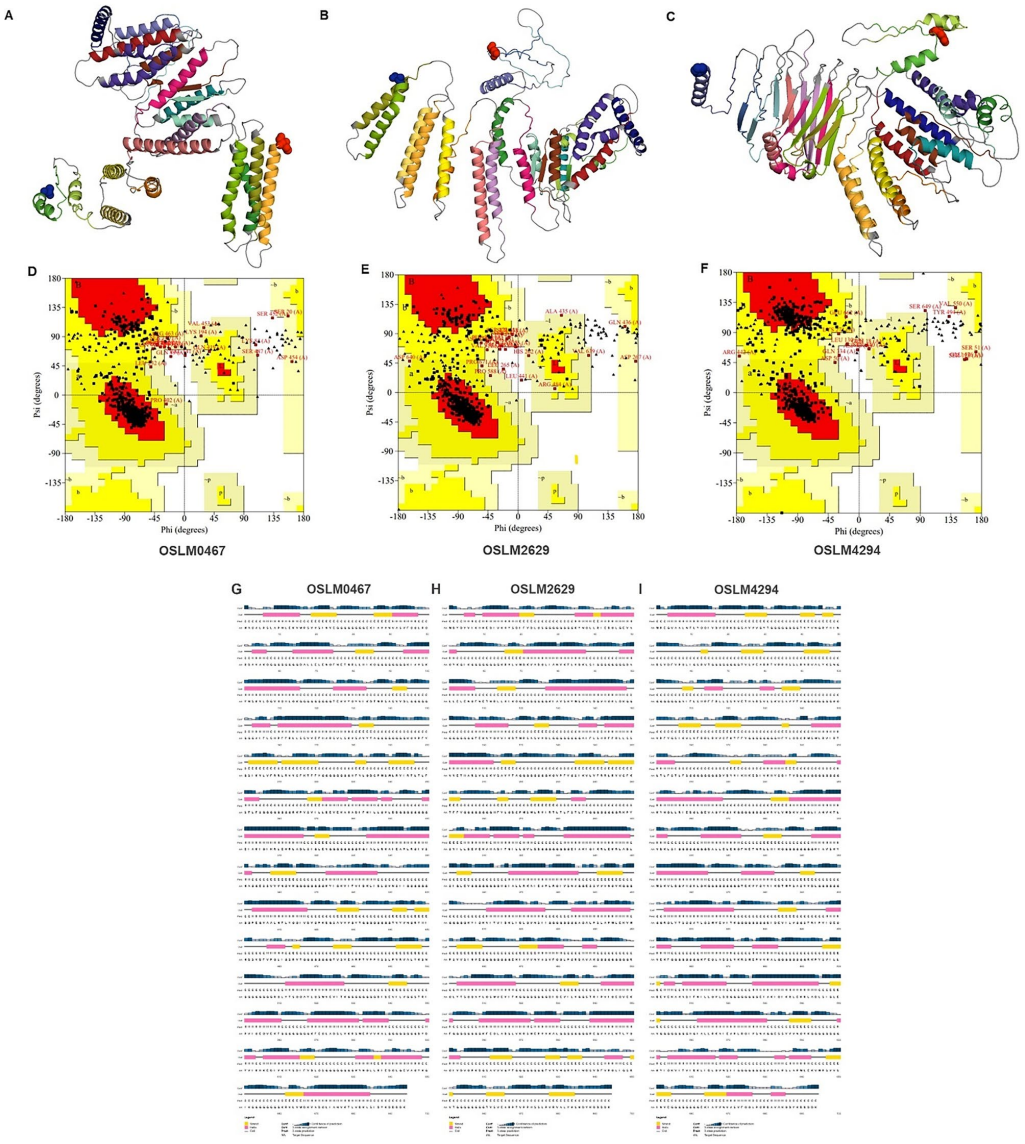
The tertiary structure, Ramachandran plot, and secondary structure prediction results of selected CMEV. [**A**–**C**] The predicted tertiary structure of OSLM0467, OSLM2629, and OSLM4294 as determined by the AlphaFold2 server. Blue and red spheres indicated the N-terminus and C-terminus within the structures, grey color indicated the linker (G_8_), and other district colors represented each joining epitope. [**D**–**F**] The Ramachandran plots of OSLM0467, OSLM2629, and OSLM4294 models showing the amino acid residues located in the most favored regions (red area), additional allowed regions (bright yellow area), generously allowed regions (light yellow area), and disallowed regions (white area). [**G**–**I**] The secondary structure prediction of the amino acid sequence based on the PSIPRED web server. Pink, yellow, and gray color bars indicated alpha-helix, beta-strand, and coil structures, respectively.

For constructing CMEVs, the 21 representative epitopes of each pathogen were grouped, obtaining a total of 7 sets of epitopes. All those groups were randomly shuffled on the Gly_8_ linker, a flexible linker that can feasibly enhance protein folding and potentially increase the accessibility of each epitope to antibodies^35^. After shuffling, 5,040 CMEV models were generated regarding 7 factorials (7!). Predicting tertiary folding structures was achieved by determining through the AlphaFold server which generated 5 predicted PDB files from each model resulting in 25,200 different tertiary protein structures. The amino acid allowance in the tertiary structure of each PDB format was checked by Ramachandran plot profiles generated by the Procheck program. Herein, only 80 candidates of the predicted CMEVs were identified by performing amino acid residues located in the most favored region of more than 80% and the disallowed region of less than 2%. Among all the predicted models, herein, three desirable candidates designated as OSLM0467, OSLM2629, and OSLM4294 were selected. The schematic diagram demonstrated that the chosen candidates contained different patterns of epitope arrangement (Fig. 1) and revealed varied structures of 3D folding (Fig. 2A–C). From the Ramachandran plot profile, the OSLM0467 showed the best allowed amino acid residues located in the most favored regions and disallowed regions at 84.6% and 1.0%. Meanwhile, OSLM2629 showed 83.25% and 1.0%, whereas OSLM4294 represented 82.8% and 0.8% (Fig. 2D–F).

### Physiochemical characterization and glycosylation determination

To evaluate the physical and chemical properties of the designed CMEV candidates, several computational analytical tools were employed. Regarding the ProtParam server, the selected CMEV constructs had predicted molecular weight (MW) of approximately 69.55 kDa and theoretical pI of 8.84. The total number of negative charges (Asp + Glu) and positive charges (Arg + Lys) were 60 and 66 residues. Importantly, the estimated half-life of the expressed CMEVs in mammalian reticulocytes (in vitro), yeast (in vivo), and *E. coli* (in vivo) were predicted as 30 h, > 20 h, and > 10 h, respectively. Their aliphatic index of 88.65 and GRAVY score of -0.057 were computed indicating the designed constructs to feasibly be thermostability and hydrophilicity. Besides these, the stability limit of OSLM0467, OSLM2629, and OSLM4294 (56.35, 56.26, and 56.96, respectively) was determined by the instability index. Since antigen post-translational modification also promotes auxiliary specificity sources for immune response^36^, thus, the glycosylation of designed CMEVs was also determined. In this study, OSLM0467 and OSLM2629 exhibited one potential *N*-glycosylation at N^76^ and N^129^, respectively. Meanwhile, OSLM4294 was predicted to possess 2 glycosylated sites of N^50^ and N^325^ under the default threshold (0.5) of the NetNglyc server. Besides* N*-glycosylation, several *O*-linked glycosylation was detected in the CMEVs. OSLM0467 potentially contained 8 positions of *O*-glycosylated site (O^181^, O^350^, O^463^, O^466^, O^475^, O^477^, O^483^, and O^488^), while OSLM2629 (O^214^, O^383^, and O^652^) and OSLM4294 (O^65^, O^475^, and O^578^) were comprised of 3 possible positions being higher than the 0.5 threshold score as determined by the NetOGlyc tool.

### Evaluation of conformational B-cell epitope, allergenicity, and antigenicity

**Figure 3:**
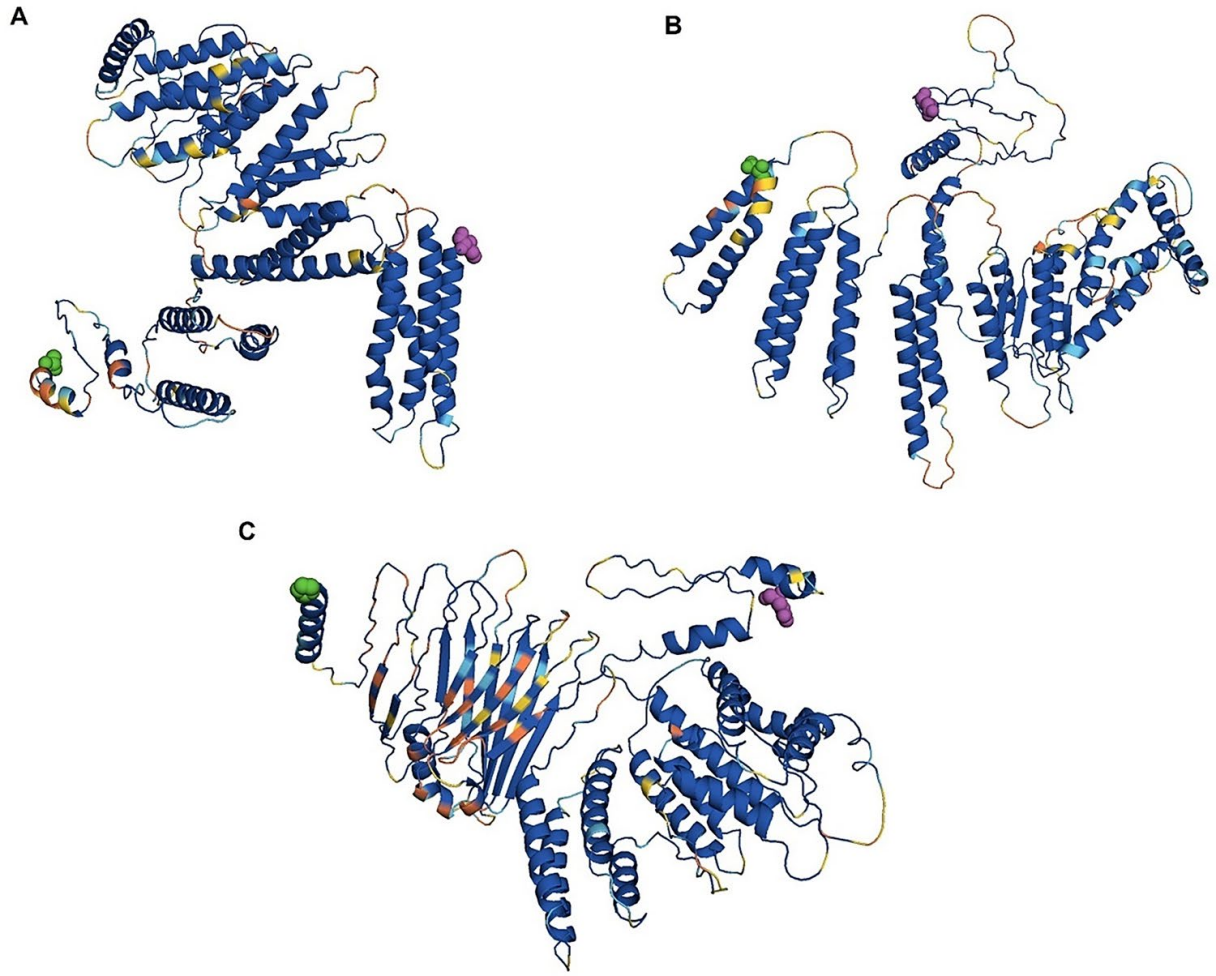
Conformational B-cell epitope prediction of the tertiary CMEV structures using Discotope 3.0. [**A**–**C**] Predicted B-cell epitope of OSLM0467, OSLM2629, and OSLM4294. The green and magenta spheres indicated the N- and C-terminus of CMEVs. Residues with the thresholds of low, moderate, and high epitope propensity were colored in light blue, yellow, and orange, respectively.

After designing the CMEV candidates by connecting the predicted linear B-cell epitope with the linker, their computed tertiary protein structures were submitted to the DiscoTope 3.0 server for identifying the B-cell epitope conformation. Three calibrations of high (1.50), moderate (0.90), and low (0.40) confidence thresholds were set for the epitope validation. Remarkedly, numerous amino acid residues within the CMEV proteins were listed as conformational B-cell epitope residues both on the selected linear B-cell fragments and the specific linkers. OSLM0467 demonstrated 48, 93, and 160 residues to be the conformational B-cell epitope when computed at high, moderate, and low thresholds, respectively (Fig. 3A). Meanwhile, OSLM4294 was listed at 51, 101, and 160 predicted residues (Fig. 3C). Notably, OSLM2629 contained the highest numbers of 62, 107, and 171 amino acids existing as B-cell epitope residues (Fig. 3B) as compared with other selected CMEVs (Fig. 3). Besides this, the antigenicity prediction by the ANTIGENpro and Vaxigen servers indicated that the CMEVs provided great scores to be antigenic substances for both bacterial and viral models. In addition, these CMEVs presented non-allergen properties regarding the AllerTOP server.

### Secondary structure observation and structure refinement

Based on the secondary and tertiary structure prediction from the PSIPRED server and Alphafold databases, each designed CMEV candidate demonstrated distinct shapes – differing in the number of secondary structures that reflected the tertiary structure. OSLM0467 contained 51.8%, 2.6%, and 45.5% of alpha-helix, beta-strand, and coil (Fig. 2G), while OSLM2629 represented 54.2%, 3.1%, and 42.8% (Fig. 2H). Notably, both candidates had a dominant helix structure which covered approximately 50% of the structure. On the other hand, OSLM4294 revealed the dominant structure of coil at 46.3% followed by 36.5% of alpha-helix, and 17.2% of beta-strand (Fig. 2I). Their amino acid contents included small nonpolar, hydrophobic, polar, and aromatic plus cysteine were also predicted and the district arrangement profiles were noticed as shown in Supplementary Fig. 1.

**Figure 4:**
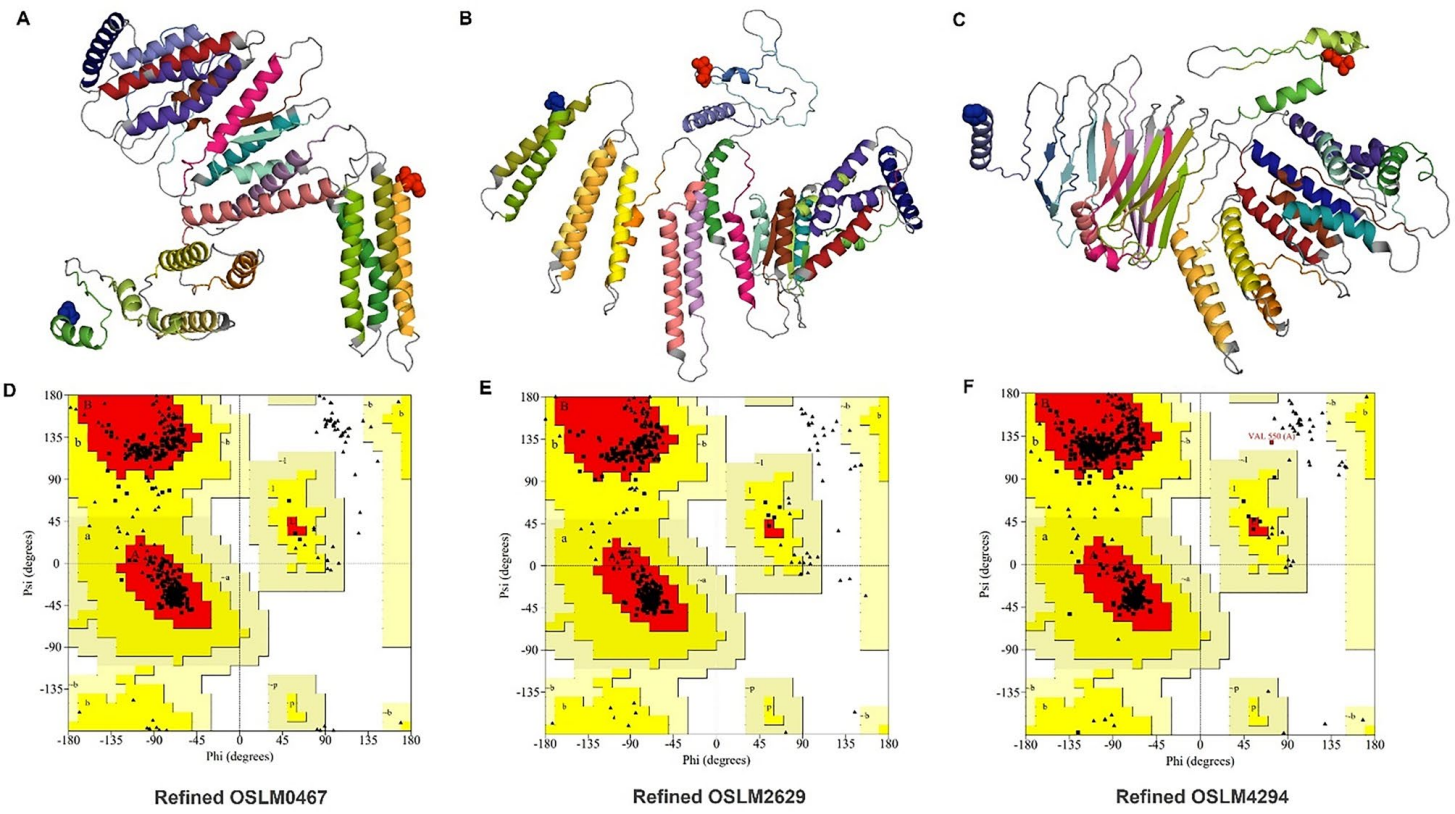
The tertiary structure and Ramachandran plot of the CMEVs after refinement. [**A**–**C**] The refined protein folding of OSLM0467, OSLM2629, and OSLM4294. Blue and red spheres indicated the N-terminus and C-terminus within the structures, while gray color indicated the linker (G_8_), and other district colors represented each joining epitope. [**D**–**F**] The plots of the refined OSLM0467, OSLM2629, and OSLM4294 models show the amino acid residues located in the most favored regions, additional allowed regions, generously allowed regions, and disallowed regions.

To obtain the precise tertiary structure of CMEVs, the predicted tertiary structure was refined by the GalaxyRefine server. In the Ramachandran plot of refined structure, amino acid residues of OSLM0467, OSLM2629, and OSLM4294, were aligned in the most favored region at 98.6%, 98.2%, and 96.7%, respectively. Surprisingly, there were no residues located in the disallowed region of OSLM0467 and OSLM2629 models, while OSLM4294 had 0.2% residues in the region (Fig. 4). Interestingly, the structure refinement indicated that greater than 96% of amino acid residues located in the favorable region which demonstrated the achievement of structure refinement.

### *In-silico* immune simulation of the CMEV constructs

**Figure 5:**
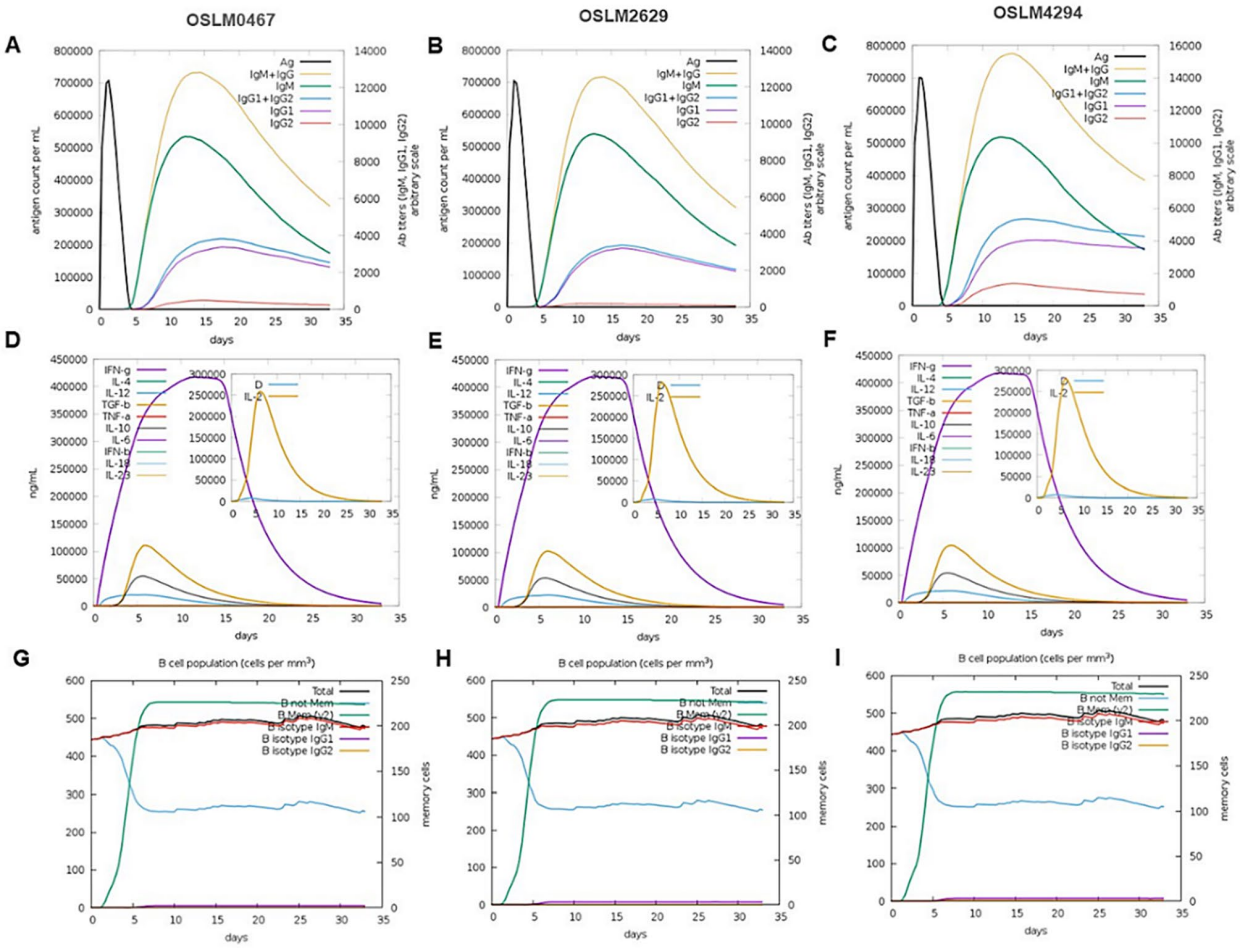
In silico simulation of immune responses after vaccination with the selected CMEVs. [**A**–**C**] The different immunoglobulin productions, while [**D**–**F**] The various cytokine inductions, and [**G**–**I**] An enhancement of the B-cell population after post-vaccination with OSLM0467, OSLM2629, and OSLM4294. The antigen was indicated in black and other immune parameters were shown in colors.

To determine the potential immune responses after vaccinating with the designed CMEVs, the *in-silico* immune simulation was performed in both terms of humoral and cell-mediated immunity. According to the simulation analysis, the antigen value peaked at approximately 7 × 10^5^ antigen count mL^−1^ and then became a baseline on day 5 after applying the vaccine. Remarkedly, immunoglobulin production including IgM and IgG was observed. The amount of IgM + IgG reached around 1.3 × 10^4^ antigen count mL^−1^ on an arbitrary scale of the 15^th^ day after exposure to OSLM0467 and OSLM2629 (Fig. 5A–B), while OSLM 4294 vaccination could promote the IgM + IgG level by almost 1.6 × 10^4^ antigen count mL^-1^ (Fig. 5C). Similarly, the OSLM4294 also showed a higher value of the PLB cell population of the IgM isotype compared with others (Supplementary Fig. 2A-C). Also, the B-cell population of isotype IgM was promoted to around 500 cells per mm^3^ and up-regulated over 30 days post-exposure. In the case of the memory B-cells, they reached the highest amount after approximately 6 days and continued after 1 month (Fig. 5G–I). Among different states of the B-cell population, the active isotype remained longer period than other types (Supplementary Fig. 2D-F). Moreover, the number of T-helper cells, cytotoxic T-cells, and NK cell populations were triggered after the selected CMEV vaccination (Supplementary Fig. 2G-P).

In addition, a great amount of interferon-gamma (IFN-γ), which is a cytokine involved with innate and adaptive immunity, was increased after CMEV application. The highest level was noted on the 10^th^ day until the 16^th^ day approximately as well and other types of simulated cytokines including TGF-β, IL-10, and IL-12 were detected (Fig. 5D–F).

### Molecular docking and molecular dynamics (MD) simulation of the selected CMEV candidates with TLR receptor

**Figure 6:**
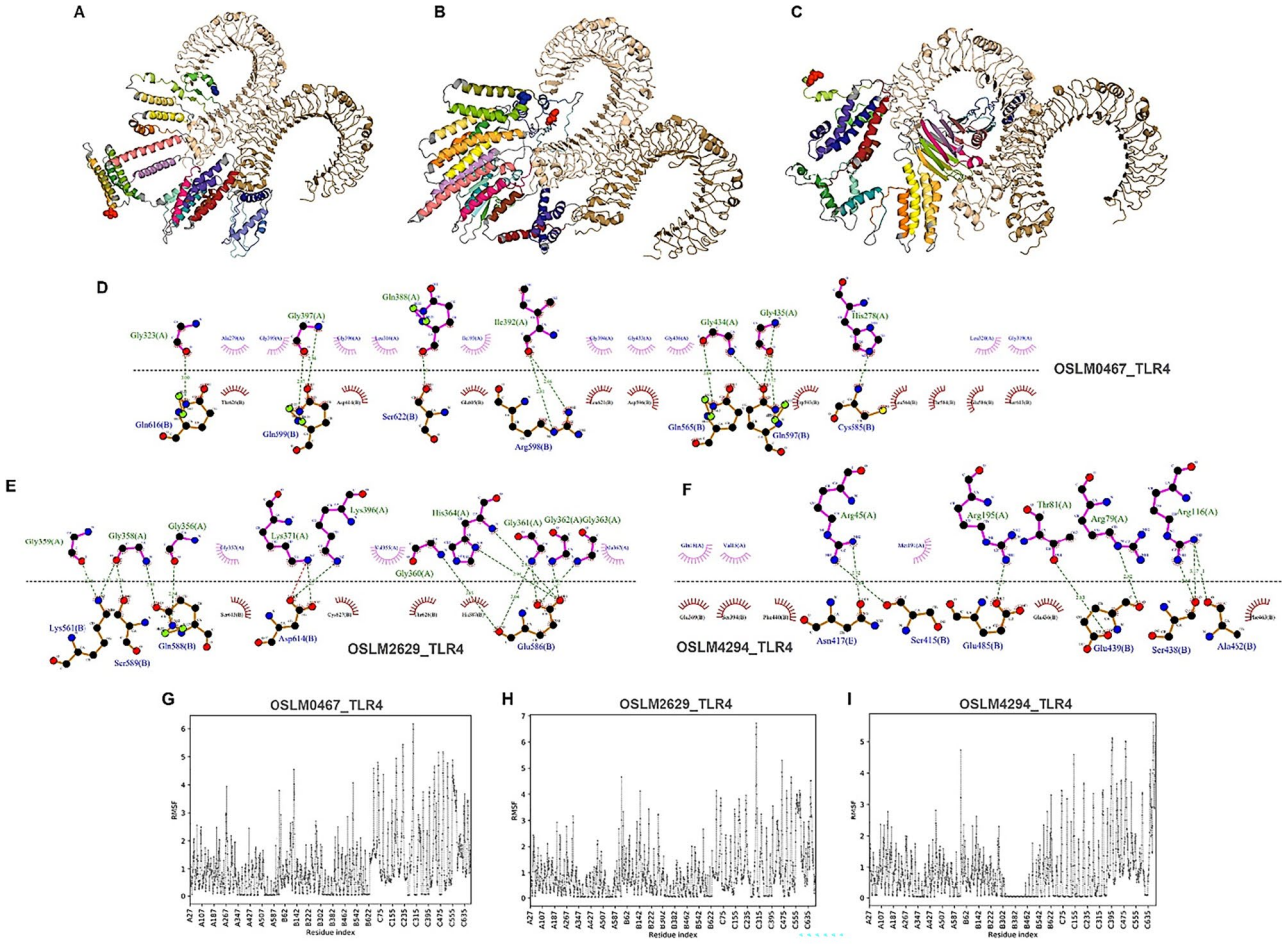
Molecular docking and MD simulation of the CMEV-TLR4 complexes. [**A**–**C**] The refined tertiary structure of OSLM0467, OSLM2629, and OSLM4294 docked with a homodimer of the TLR4 receptor by the ClusPro server. [**D**–**F**] The interacting residues of the TLR4 receptor with OSLM0467, OSLM2629, and OSLM4294 through the LIGPLOT v.2.2. Hydrogen bonds and salt bridges are shown as green and red dotted lines. Hydrophobic interactions made by the receptor and the ligand (vaccine construct) are indicated as pink and red semi-circles. [**G**–**I**] The RMSF plot of the CMEV-TLR4 complexes analyzed through the CABS-flex 2.0 server.

To verify the potential interaction of the designed CMEV constructs with the TLR receptor, the docking complexes were first performed by the Cluspro 2.0 server followed by the amino acid interaction determination by LIGPLOT v2.2. By default, the top 10 models of the docked complexes were displayed. Herein, the first model of OSLM0467, OSLM2629, and OSPM4294 binding with homodimeric TLR4 receptor providing the lowest docking score (indicated high-affinity binding and molecular interaction)^37^ was chosen, and the docked structure was observed on the Pymol program (Fig. 6A–C). The TLR4 docking OSLM0467 model represented the lowest energy score of -1112 kJ.mol^−1^, while OSLM2629 and OSLM4294 possessed the score of -1224.3 kJ.mol^−1^ and -1073.6 kJ.mol^−1^. Additionally, the result of the vaccine-receptor interaction diagram from the LIGPLOT demonstrated that the OSLM0467 complexes had 11 hydrogen bonds as well as 10 hydrophobic interactions made by the TLR4 receptor and the vaccine ligand (Fig. 6D). For the OSLM2629 complexes, 14 hydrogen bonds, 1 salt bridge, and 3 hydrophobic bindings of the receptor and 4 bindings of the ligand was depicted (Fig. 6E). Meanwhile, the OSLM4294 complexes exhibited 8 hydrogen bonds along with 3 and 5 hydrophobic formations for the receptor and vaccine construct (Fig. 6F). Molecular docking was used to study the interaction of each CMEV molecule and TLR4 receptor. Subsequently, the MD simulation was also performed to investigate the relation of the docked surface residues of these interacting molecules. The flexibility of the CMEV-TLR4 complexes was determined with the RMSF score through the CABS-flex 2.0 server. Most of their amino acid atoms represented the RMSF values below 4.0 Å and the average RMSF profiles in ranges of 0.81 – 1.06 Å. Notably, the RMSF score of the OSLM4294-TLR4 complex revealed the least fluctuation following OSLM0467 and OSLM2629, respectively (Fig. 6G–I; Supplementary Fig. 3). Among these three complexes, the OSLM4294—TLR4 complex demonstrated the most attached/ least fluctuation interpreting as the most stable system from the others.

### Codon optimization and in silico cloning

To study the protein expression of the selected CMEV candidates, their amino acid sequences were back-translated to the nucleotide sequences according to codon usage preference by the GeneArt™’s gene optimization standard. Herein, we focused on both bacterial host and tilapia expression systems. Therefore, the codon optimization based on the codon bias of *E. coli* and Nile tilapia (*Oreochromis niloticus 113 CDS* gene) was performed. In the optimized process, the GC regions potentially higher than 80% and lower than 30% were avoided from the optimized CMEV candidate sequences to prolong the mRNA half-life. Besides, the cis-acting sequence motifs including (1) the internal TATA-boxes, chi-sites, and ribosomal entry sites; (2) the motifs of RNA instability; (3) the splice donor and acceptor sites in higher eukaryotes; (4) the stretches of AT-rich or GC-rich sequence; and (5) the repeat sequences and RNA secondary structures were eliminated to reduce the possible negative impact on CMEV protein expression.

After optimization, OSLM0467, OSLM2629, and OSLM4294’s codon adaptation index (CAI) values were matched with *E. coli* codon utilization of 0.94, and the bias of *O. niloticus* resulted in 0.87. These CAI values supported that the optimized genes were probably expressed with high and stable rates in the optimized host. The codon quality distribution of all optimized CMEVs exhibited the sequence codon percentage that was mostly distributed in the 90–100 range at approximately 83% and 70% of *E. coli* and *O. niloticus* optimization, respectively (Supplementary Fig. 4A-C and 4G-I). Moreover, the GC content adjusted to *E. coli* codon usage of all optimized CMEV genes demonstrated the average value at 50% (Supplementary Fig. 4D-F). In contrast, the average content adapted to *O. niloticus* exhibited 58% for OSLM0467 and OSLM2629 and 57% for OSLM4294 (Supplementary Fig. 4 J–L).

**Figure 7:**
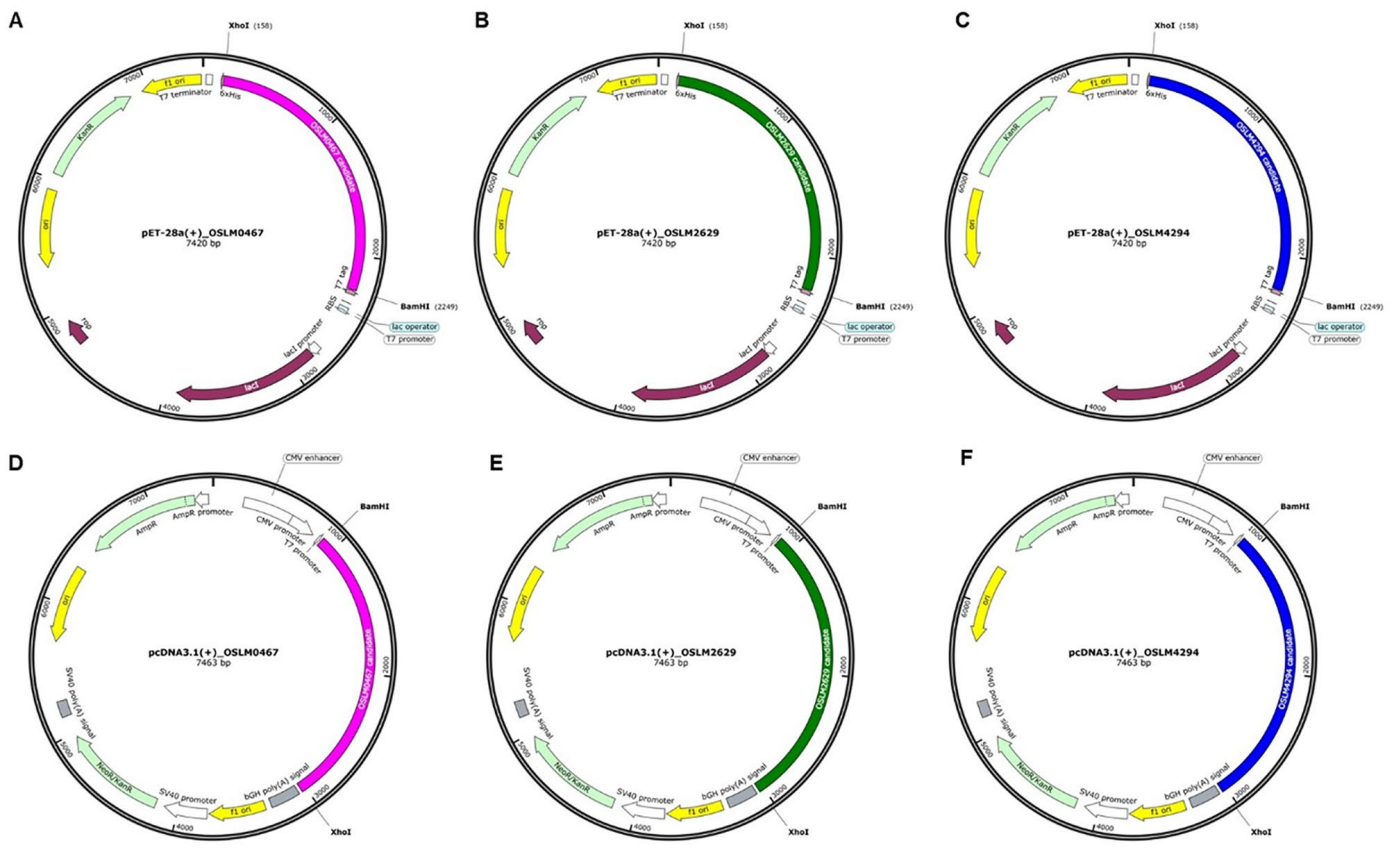
*In-silico* cloning of the CMEVs to pET-28a( +) [**A**–**C**] and pcDNA3.1( +) [**D**–**F**] vectors. The pink, green, and blue regions represented the inserted CMEV nucleotide sequence of OSLM0467, OSLM2629, and OSLM4294, respectively.

Additionally, single-stranded RNA folding of the optimized CMEVs was demonstrated regarding the minimum free energy (MEF) structure. Notably, the 5’-end of their RNA structures was free from pseudo-knot and hairpin loop structures (Supplementary Fig. 5). The free energy of the thermodynamic ensemble for OSLM0467, OSLM2629, and OSLM4294 was -836.22,

-860.25, and -836.33 kcal mol^-1^, respectively. At last, the synthesized genes of the optimized CMEVs would be inserted into pET28a ( +) and pcDNA3.1( +) vectors for further study in protein expression of both *E. coli* and tilapia cell line systems (Fig. 7).

## Discussion

In the past, vaccine development generally relied on conventional vaccinology by starting from cultivating pathogenic microorganisms, subsequently identifying antigens, and testing immunogenicity until studying vaccine efficacy in animals. Though there are a number of successful vaccines including attenuated vaccines, inactivated vaccines, and subunit vaccines developed regarding the traditional approach, this strategy might be not suitable for some vaccine productions such as bivalent and multivalent vaccines. One of the crucial factors for producing inactivated polyvalent vaccines is controlling the quality of bacterial vaccine seeds^10^. Moreover, there are still many challenges that need to be critically concerned in developing multivalent vaccines. For instance, the production of those vaccines based on the conventional method probably increases manufacturing costs and times, antigen impurities, reactogenicity, quality control, and storage costs. Likewise, it feasibly limits the composition and antigenicity of utilized antigens^38^. Importantly, the multiple injections of each specific monovalent vaccine are inconvenient, particularly for small fish^39^, so the potent polyvalent vaccine with a single vaccination needs to be more comprehensive and developed.

To surpass the hindrances of the traditional approach, recently, the concepts of reverse vaccinology and structural vaccinology have been dramatically introduced in generating novel potential vaccines for both human and veterinary disease prevention. This advanced method benefits in searching the potentially immunogenic targets within the pathogenic genomes before identifying the feasibly antigenic epitopes for developing the promising next-generation vaccine platform, which is the multiepitope-based vaccine^40^. In this regard, we applied an innovative strategy based on the vaccinomics approach to design the polyvalent multiepitope vaccine against the seven causative agents of tilapia fish. Under the previous study, the designed multiepitope vaccine based on the B-cell epitopes could stimulate antibody production and potentially enhance the survival rate of the vaccinated fish against *S. agalactiae* infection^13^. Also, another publication revealed that the flounder fish vaccinated with the recombinant B-cell multiepitope vaccine exhibited a significant increase in their immune responses (such as IgM, CD4, and CD8) and resulted in higher RPS rate (74%—78%) against *S. iniae* than the FKC (48%)^41^. Consequently, in this recent study, a number of literate vaccine candidates were chosen and the potential immunodominant B-cell epitopes were subsequently analyzed through multiple bioinformatic servers. Since the predicted conserved epitopes are probably responsible for broad-spectrum protection among the district genus and species of pathogens^42^, the consensus epitope sequences among different species were considered for the vaccine construction. Herein, only the potential 21 predicted B-cell epitopes were selected and shuffled with the flexible linker of G_8_ to generate the novel heptavalent chimeric vaccines. In addition, the structural determination based on in silico analysis was responsible for predicting the secondary and tertiary structures, physiochemical characteristics, and other properties including Ramachandran profile, antigenicity, allergenicity, and post-translational modification.

Evidently, several publications demonstrated that the BcePred, BepiPred, ABCpred, COBEpro, IEDB, SVMTriP, and LBtope servers served as examples of productive tools to identify the efficacious linear B-cell epitopes for designed multiepitope vaccine against antibiotic-resistant bacteria and other severe pathogens extensively^43,44^. However, various types of specific linkers including EAAAK, GPGPG, GGGGGGGG, GGGGS, and AAT have been utilized to separate the adjacent epitopes or peptides in recombinant vaccines. The linker option can probably affect protein folding, flexibility, and stability, as well as possibly facilitate the immunorecognition of the constructed immunogenic epitopes^35,43^.

Among 25,4000 models of our designed chimeric multiepitope vaccine (CMEV), only 80 CMEV models carried more than 80% of amino acid residues located in the most favored region and less than 2% located in the disallowed region of Ramachandran plot prediction. After submitting for physiochemical characterization, glycosylation, allergenicity, and antigenicity determination, all of them were represented in similar profiles for physiochemical, allergenic, and antigenic features, but only 18 CMEV models provided a high potential threshold score of glycosylation prediction. Among them, the selected CMEV candidates of OSLM0467, OSLM2629, and OSLM4294 were listed in the top 3 of the best Ramachandran plot profiles by showing high amino acid residues located in the most favored region and low amino acid residues located in the disallowed region. Also, these CMEVs demonstrated the district compositions at the secondary structure level. Thus, we first chose these three candidates for other analyses and synthesized their optimized nucleotide sequences further to verify their protein expression characteristics and their vaccine effectiveness.

From the ProtParam prediction, the CMEVs represented an instability index score greater than 40 which was probably categorized as unstable protein. However, regarding the structural determination and refinement, all of the refined CMEVs demonstrated high-quality protein structure regarding most of the amino acid residues deposited in the most favored regions and disallowed regions in the range 96.7–98.6% and 0.0–0.2% in the Ramachandran plot in agreed with Wlodawer’s definition^44^. By Zhao et al. study^45^, their predicted tertiary structures demonstrated the excellent quality of protein folding, but the proteins still performed instability based on the index. This instability might lead to protein auto-processing that feasibly provides immunogenicity benefits by assisting antigen processing for presenting to immune cells^46^. According to allergenicity and antigenicity evaluation, the chosen CMEVs were classified as non-allergens^47^ and excellent antigenic substances^48^. Moreover, the post-translation modified antigens are probably involved with its immunomodulatory role^36^. The major type of protein modification is glycosylation, so this profile of the CMEVs was also considered. Herein, all of the selected CMEVs represented several predicted sites of *N*-linked and *O*-linked glycosylation. Notably, almost crucial molecules related to innate and adaptive immune recognitions are represented as glycoproteins^49^. This evidence supports that our predictably glycosylated CMEVs might influent antibody activation and production.

The activation of specific immunity through B-cell, receptors aligned on the surface of B-cell are recognized by both linear and conformational B-cell epitopes. Thus, herein, apart from the linear epitope determination, the conformational epitopes were examined using computer-aided analysis. According to the DiscoTope 3.0 server, the reliable tool for validating the B-cell epitopes of tertiary structure protein^50^, the numerous amino acid residues of the refined CMEVs’ tertiary structure were potentially designated as the conformational B-cell epitope residues. In particular, not only the predicted conformational B-cell residues located in the selective linear B-cell fragments but also noticing them greatly involved in the specific linkers. This finding feasibly supported that our designed CMEV candidates could stimulate humoral immune response as an immunoglobulin M (IgM), which is dominant in the adaptive immune system for preventing fish pathogenic infection^51^. Correspondingly, the immune simulation result regarding the immunoinformatic approach also evidenced that the constructed CMEVs could hugely induce the immunogenic responses of both cellular and humoral immunity. The concept of computation-based simulation in identifying the effective vaccine candidates was obviously proved by in vivo trials in sea bass against *Listonella anguillarum* and *Photobacterium damselae*^52^.

Additionally, protein–protein docking was introduced for studying the binding of the CMEVs and the immune receptors. Herein, the vaccine constructs were capable of clearly interacting within the pocket of the TLR4 receptors through several hydrogen bonds, salt bridges, and hydrophobic interaction. As the previous reports on designing the epitope-based vaccine against *Vibrio harveyi* and TiLV, the Toll-like receptor4 (TLR4) receptors were docked with the CMEVs to confirm the ligand-receptor interaction^11,14^. The TLR signaling pathway is significant in fish's innate immune response against infectious pathogens. Among a variety of TLR receptors, TLR2, TLR4, TLR5, and TLR9 are supposed to be recognizers of the bacterial ligands^15^. Recently, TLR4 has been reported in several fish species including channel catfish, grass carp, common carp, mrigal, zebrafish, rare minnow, and seabream. Previous studies evidenced that the TLR4 receptor potentially responds to pathogenic PAMPs including bacteria, fungi, viruses, and protozoa^14,53^. Hence, in the current study, this receptor was employed as a representative of the immunogenic receptor in the molecular docking determination. Correspondingly, several publications on the multiepitope vaccine-based computational design that represents potential docking with TLR receptors could induce antibody production after in vivo immunization^54,55^. Moreover, the RMSF profiles of the CMEV-TLR4 complexes with an average score of ≤ 4 Å might define their complex stability resulting in stimulating receptor response by fluctuating residues^11,14–56^.

Lastly, the codon optimization of their nucleotide sequences based on *E. coli* and *O. niloticus* codon bias was conducted. Notably, all the optimized sequences showed the perfect scores for all concerned parameters including the negative cis-acting sites, GC content, and codon adaptation index (CAI)^57^. These presumed that the optimized CMEV candidates will perform high and stable expression rates in the adapted hosts. Taken together, using reverse vaccinology and structural vaccinology using in silico design concepts is beneficial to identify the efficacious immunogenic components and help to accelerate the developmental timeline of the novel vaccine candidates. However, validation of the vaccine candidates based on the computer-aided design needs to be further studied in vitro and in vivo to confirm the vaccine efficacy against the target pathogens.

## Materials and methods

### Immunogen selection and B-cell epitope identification

In this study, the immunogens of seven pathogens including *E. tarda*, *F. columnare*, *F. noatunensis*, *S. iniae*, *S. agalactiae*, *A. hydrophila,* and TiLV were selected based on high-feasibility potential vaccine candidates from the previous reports. Amino acid sequences of the chosen immunogens were retrieved from the National Center for Biotechnology Information (NCBI) protein database and aligned to find consensus sequences among several species. Subsequently, the antigenic peptides and B-cell epitopes were predicted on the conservancy sequence of the selected immunogens using three online servers including the BcePred Prediction Server^58^, Antigenic Peptides program^59^, and IEDB server^60^. Furthermore, hydrophobicity and secondary structure of predicted epitopes were evaluated using the Peptide 2.0 server and JPred server^61^, respectively. The required epitopes chosen for the further chimeric multiepitope vaccine (CMEV) construction would meet all of the following criteria: 1) exhibiting similar conserving epitope regions as predicted by 2–3 epitope prediction servers 2) containing at least 19 amino acid residues, 3) carrying low hydrophobicity, and 4) demonstrating alpha-helix structure.

### Chimeric multiepitope vaccine (CMEV) construction and molecular modeling

To construct the CMEVs, all predicted linear B-cell epitopes were assembled and linked together with a glycine linker (Gly_8_)^35^. The selected epitopes were randomly shuffled to formulate the CMEV candidates. The AlphaFold2 server rendered the tertiary structure regarding multiple sequence alignments of MMseqs2^62^. The predicted tertiary models were subsequently submitted to the PROCHECK program v.3.5.4 to evaluate the stereochemical quality of the protein structures based on Ramachandran plot analysis by revealing the location of their amino acid residues in four possible regions including the most favored-, additional allowed-, generously allowed-, and disallowed regions^63^.

### Physiochemical characterization and glycosylation determination

Regarding the Ramachandran plot profile, only the CMEV tertiary models with more than 80% of amino acids aligned in the favored region and less than 2% aligned in the disallowed region were selected for physiochemical characterization and glycosylation determination. The selected CMEV amino acid sequences were submitted to the Expasy Protparam tool^64^ to compute their physiochemical characteristics including the molecular weight (MW), theoretical pI, amino acid composition, estimated half-life, aliphatic index, instability index, and grand average of hydropathicity (GRAVY). In addition, the glycosylation sites were assessed using the NetNGlyc 1.0 server^65^ and NetOGlyc 4.0 Server^66^.

### Evaluation of conformational B-cell epitope, allergenicity and antigenicity

Apart from using the sequence-based approach in the B-cell epitope prediction, the structure-based method was also performed to demonstrate the direct accessibility of B-cell receptors to antigen tertiary structure. Herein, the conformational B-cell epitope residues on the tertiary CMEV construct were predicted by the DiscoTope 3.0 server, utilizing an inverse folding performance of the predicted structures based on the present state-of-the-art algorithms^47^. Allergenicity of the selected CMEVs was determined by the AllerTOP server using the database of variant-collected antigens including 684 foods, 1,156 inhalants, and 555 toxin allergens^44^. In addition, the overall allergenicity of recombinant CMEVs was evaluated by the ANTIGENpro server^67^ and Vaxigen v2.0 server^68^.

### Secondary structure observation and structure refinement

The PSIPRED web server, depending on two feed-forward neural networks of the Position-Specific Iterated–BLAST algorithm (PSI-BLAST), was used to determine the secondary structure of the CMEVs^69^. Additionally, the secondary structure compositions were performed by the 2Struc server comparing district secondary structure assignment algorithms^70^. The refinement of the predicted tertiary structure was conducted by the GalaxyRefine server to improve the model structure quality depending on the CASP10 assessment^71^. For further estimating the quality of their tertiary structures, the Ramachandran plot of the refined CMEV models was regenerated by the PROCHECK program.

### In silico immune simulation of the CMEV constructs

The selected CMEVs were submitted to the C-IMMSIM online server^72^ to predict the immune response and immunogenicity after vaccination. Herein, cell-mediated and humoral immune response profiles including T-cell, B-cell, NK-cell, cytokine, and immunoglobulin were verified based on the default parameters and simulation steps.

### Molecular docking and molecular dynamics simulation of the selected CMEVs with TLR receptor

To elucidate the interaction between CMEV and TLR receptors, the protein–protein docking was conducted by the ClusPro web server^73^ to verify the complex interaction and affinity. Herein, the selected CMEV candidates were docked with TLR4 homodimer receptor (PDB ID: 4G8A) followed by evaluating their energy structure. Subsequently, the predicted interactions of the CMEV-receptor complex including salt-bridge, hydrogen bond, and hydrophobic interaction were performed using the LIGPLOT software^74^. The CABS-flex 2.0 server^75^ was employed with the default parameters to preliminary study the molecular dynamic simulations by predicting the stability of the root mean square fluctuation (RMSF) of the CMEV-receptor complex.

### Codon optimization and in silico cloning

The amino acid sequences of the selected CMEVs were back-translated to DNA nucleotide sequences based on two codon usages, *E. coli* and tilapia (*Oreochromis niloticus 113 CDS*). The codon adaptation index (CAI) of the constructs was carried on by GeneArt™’s gene optimization process. In addition, the optimized sequences were attached with double restriction enzymes of *BamH*I and *Xho*I at 5’- and 3’- end before being synthesized by GeneArt® Gene Synthesis (Thermo Fisher Scientific, USA)^57^. The *E. coli* and tilapia optimized sequences were inserted into the pET-28a ( +) vector and pcDNA3.1 ( +) vector for further studying the recombinant CMEV protein expression in prokaryotic and eukaryotic systems, respectively. In silico cloning of the optimized CMEVs in the expression vectors was revealed by the SnapGene software (www.snapgene.com). Additionally, the optimized DNA sequence of CMEVs was transcribed to RNA sequence before further determining the secondary structure folding and the thermodynamic ensemble energy by the RNAfold web server^76^.

### Supplementary Information

Supplementary Information.

## Data Availability

The datasets generated and/or analyzed during this study are available from the corresponding author upon reasonable request.
