# Supplementary material for: Computational design of novel chimeric multiepitope vaccine against bacterial and viral disease in tilapia (Oreochromis sp.)
Source: Sci Rep. 2024 Jun 18;14:14048. doi: 10.1038/s41598-024-64383-z (PMC11189486; doi:10.1038/s41598-024-64383-z)

**Computational design of novel chimeric multiepitope vaccine against bacterial and viral disease
in tilapia (*Oreochromis* sp.)**

Ansaya Pumchan^1,2^, Porranee Proespraiwong^1,2^, Orathai Sawatdichaikul^3^, Thararat Phurahong^1,2^,
Ikuo Hirono^4^, and Sasimanas Unajak^1,2*^

**Supplementary information**

**Supplementary Table 1** Summary of all identified B-cell epitopes for constructing CMEV candidates.

| **Pathogens  (No. of total epitope)** | **Genes (No. of total epitope)** | **Protein name** | **Epitope sequence** | **Secondary structure** | **Length (amino acid residues)** | **No. of analyzed program** | **RPS (%)** | **Accession No.** | **Reference** |
| --- | --- | --- | --- | --- | --- | --- | --- | --- | --- |
| *E. tarda*  (13) | *DegP* (2) | Serine endoprotease | AQALPSLAPMLEKVMPSVVSINVE | ------HHHHHHHHHHHHEEEE-- | 24 | 3 | 89.0 | PVD94192.1 | 16 |
|  |  |  | VPSQFQQFFGEDSPLCQPGSP | -----HHH------------- | 21 | 2 |  |  |  |
|  | *Eta2* (1) | Skp-like molecular chaperone | EKIAVVNVASVFQQLPQRDAVAKQ | --EEEEEHHHHHHH---------- | 24 | 3 | 83.0 | ADR31356.1 | 17 |
|  | *Esa1* (5) | Putative surface antigen | LEDFYYSVGKYNATVKAVVTPLPR | ---HHH------EEEEEEEE---- | 24 | 3 | 79.0 | ADK94171.1 | 18 |
|  |  |  | RVDLKLVFTEGKSAQIQQINIVGNH | --EEEEEEE-----EEEEEEE---- | 25 | 3 |  |  |  |
|  |  |  | IKQLLGRYGYAYPCVQTQPE | -HHHHHH------------- | 20 | 3 |  |  |  |
|  |  |  | KDKTVVLHMNIDAGNRYYVRQIRFV | ---EEEEEEEE-----EEEEEEEE- | 25 | 3 |  |  |  |
|  |  |  | RTGLGYVHNDLSDMQPQVAMW | ----------------EE--- | 21 | 2 |  |  |  |
|  | *OmpA* (2) | Outer membrane protein A | KAQGIQLAAKLSYPLMDNLDVYTRL | ---EEEEEH---------------- | 25 | 2 | 55.0 | GAC63059.1 | 19 |
|  |  |  | KPRAALINCLAPDRRVVIEVKGI | -----EE-------EEEEEEE-- | 23 | 3 |  |  |  |
|  | *GapA* (1) | Glyceraldehyde-3-phosphate dehydrogenase A | TDEAVVSTDFNGEVCTSVFDA | ---EEE-------HHHHHH-- | 21 | 3 | 71.4 | SPW31014.1 | 20 |
|  | *Eta21* (2) | Putative peptidase | KGSVNLSFNCQVQYQRSQCAIHLET | ---EEEE--EEEEEE---EEEE--- | 25 | 3 | 69.0 | ACY64541.1 | 16 |
|  |  |  | SQQSGVVDIAPEAYQKLRQTFLSALTL | -----EEE--HHHHHHHHHHHHH---- | 27 | 2 |  |  |  |
| *F. columnare*  (9) | *OmeP* (3) | Outer membrane efflux protein precursor | KLTLSQLIFDGSYLVGLESAKTYLKI | --HHHHHH----EEE-----EEEE-- | 26 | 3 | 68.0 | ANO47148.1 | 21 |
|  |  |  | YGDVLLAQESIMIVERNKVILEKTL | ---HHH----EEEEE---EEE---- | 25 | 2 |  |  |  |
|  |  |  | RQLYTSQQAYLQSMVEVITK | ---HHHHHHHHHHHHHHH-- | 20 | 3 |  |  |  |
|  | *DnaK* (6) | Molecular chaperone DnaK | TAAALAYGLDKAAHDKKIAVYDL | -HHHHHHH---------EEEE-- | 23 | 2 | Increase survival rate > 30% | PDS21884.1 | 22 |
|  |  |  | TEINLPYITATASGPKHLVQTLT | ------EEEE------EEEEE-- | 23 | 3 |  |  |  |
|  |  |  | KFEQLADSLVKRSMEPVVKAL | --HHHHHHHHHHHHHHHHH-- | 21 | 2 |  |  |  |
|  |  |  | DIDEVILVGGSTRIPVIQEQVEKF | ---EEEEE------HHHHHHHH-- | 24 | 3 |  |  |  |
|  |  |  | RKSQVFSTAVDNQPSVEIHVLQGE | ----EEEE-------EEEEEE--- | 24 | 3 |  |  |  |
|  |  |  | HLDGIPPAPRGVPQIEVTFD | -------------EEEEE-- | 20 | 3 |  |  |  |
| *F. noatunensis*  (20) | *ClpB* (7) | ATP-dependent chaperone ClpB | NNPVLIGEPGVGKTAIVEGLAQR | ---EEE------HHHHHHHHH-- | 23 | 2 | No data | PLR04593.1 | 23 |
|  |  |  | RLKSVLKELSKQEGNVILFIDEL | -HHHHHHHHHH----EEEEE--- | 23 | 3 |  |  |  |
|  |  |  | RFQKVLVDEPTVEDTIAILRG | ---EEEE-------HHHH--- | 21 | 3 |  |  |  |
|  |  |  | SFLHKRVIGQDQAIKAVSNA | --HHHHH-----HHHHH--- | 20 | 2 |  |  |  |
|  |  |  | RKPYSVILLDEVEKAHADIFNILLQVLDD | ----EEEEE--HHH-HHHHHHHHHH HH-- | 29 | 3 |  |  |  |
|  |  |  | EIAKIQIKRLEKRLADLSIGLEV | --HHHHHHHHHHHHHH------- | 23 | 2 |  |  |  |
|  |  |  | ENPLALKLLDGEFKAEDKIVVDID | -----EEEE---------EEEE-- | 24 | 2 |  |  |  |
|  | *GroEL* (6) | Molecular chaperone GroEL | VNTLANAVKVTLGPKGRNVVLDKSF | --HHHHHHHHH------EEEEE--- | 25 | 3 | 22.0-50.0 | ALK94214.1 | 24 |
|  |  |  | ARLVEELKALSKPCSDPKSIEQVGT | -HHHHHHHHH---------H----- | 25 | 2 |  |  |  |

**Supplementary Table 1 (cont.)** Summary of all identified B-cell epitopes for constructing CMEV candidates.

| **Pathogens  (No. of total epitope)** | **Genes (No. of total epitope)** | **Protein name** | **Epitope sequence** | **Secondary structure** | **Length (amino acid residues)** | **No. of analyzed program** | **RPS (%)** | **Accession No.** | **Reference** |
| --- | --- | --- | --- | --- | --- | --- | --- | --- | --- |
| *F. noatunensis*  (20) | *GroEL* (6) | Molecular chaperone GroEL | DELDVVEGMQFDRGYLSPYF | ----EE---E---------- | 20 | 2 | 22.0-50.0 | ALK94214.1 | 24 |
|  |  |  | ENPYILIVDKKISNIRDLLPILEGV | ---EEEEEE--H-HHHHHHHHHH-- | 25 | 3 |  |  |  |
|  |  |  | LATLVVNNMRGVVKVCAVKAP | HHHHHH-----EEEEEE---- | 21 | 3 |  |  |  |
|  |  |  | HGIALLRKAIEAPLRQIVSNAGGESSVVVNQVK | --HHHHHHHHHHHHHHHHHH----HHHHHH--- | 33 | 3 |  |  |  |
|  | *IglB* (3) | Intracellular growth locus protein B | KYIQKVITVIDKLIDLQVNSII | -HHHHHHHHHHHHHHH------ | 22 | 3 | No data | ABK90199.1 | 23 |
|  |  |  | WLKVQEVCQEDYDNVEVSILDVK | ---HHHHH------EEEEEEE-- | 23 | 3 |  |  |  |
|  |  |  | SNACFFSVNSAKKVEEFVDG | ---EEEE----HHHHHHH-- | 20 | 2 |  |  |  |
|  | *OmpA* (4) | Outer membrane protein A | QYNQLVGRVFAGLGEGVVNF | --HHHHHHHHHHH------- | 20 | 3 | No data | KXO64237.1 |  |
|  |  |  | AAQTVAMPTIDESKYVLPAGIKQ | ---EEE--------EEEE----- | 23 | 2 |  |  |  |
|  |  |  | EDGVACYTVNGDDVTVYLDTK | ---EEEEEE----EEEEE--- | 21 | 3 |  |  |  |
|  |  |  | KAIASFVNFIKDSNISSVTVKGY | -HHHHHHHHHH-----EEEEE-- | 23 | 2 |  |  |  |
| *S. iniae* (14) | *SagH* (2) | Streptolysin S export transmembrane protein SagH | LINMLVTNYVDNAKTYDSIVKLYPQT | -HHHHHHHHH-----HHHHHHH---- | 26 | 2 | 80.0-92.6 | EKB52946.1 | 25 |
|  |  |  | LINQLSHISPIKWVNDSLFFIIFG | -----------EE----EEEEEE- | 24 | 3 |  |  |  |
|  | *Sia10* (1) | Putative secretory protein Sia10 | KAGSILQSSAGGYGHVAYVESVGS | ----EEE------EEEEEEE---- | 24 | 2 | 73.9-92.3 | ADJ51067.1 | 18 |
|  | *Pdha1* (2) | Pyruvate dehydrogenase (acetyl-transferring) E1 subunit alpha | NTPHLYTRAEAYGIPGFYCED | -----------------E--- | 21 | 3 | 66.7-74.1 | ESR10610.1 | 26 |
|  |  |  | YGAVLALDKKDVFAPTYRDVFPGVKF | ---EEE----------HHHHHH---- | 26 | 2 |  | OHX26599.1 |  |
|  | *Sip11* (3) | Periplasmic_Binding_Protein_Type_2 | ASKPVADIVFGGSYTQYAQHPSLFEK | -------EEE---------------- | 26 | 2 | 69.7 | ADE61838.1 | 27 |
|  |  |  | ANIKVIYPEEGSVFLPASAA | --EEEEE-----EEEE---- | 20 | 3 |  |  |  |
|  |  |  | DYDYVIKHKEDIVKHYSDIFVDLQS | ---EEHH-HHHHHHHHHHHEEE--- | 25 | 3 |  |  |  |
|  | *Eno* (3) | Enolase | SIITDVYAREVLDSRGNPTLEVEVYT | ---HHHHHHHHH------EEEEEEE- | 26 | 3 | 63.0 | EKB52954.1 | 3 |
|  |  |  | WGAEVFHALKKILKERGLVTA | ---HHHHHHHHHHHH------ | 21 | 2 |  |  |  |
|  |  |  | KYNQLLRIEDQLGEVAQYKGIK | ---HHHHHHHHHHHH------- | 22 | 2 |  |  |  |
|  | *Gapdh* (3) | Glyceraldehyde-3-phosphate dehydrogenase | ELGSVPAMTTQEVDFVYASA | -------------EEEE--- | 20 | 2 | 63.0-77.8 | RLU98986.1 | 26 |
|  |  |  | KRVLVMDKVADQLIANVKTLVDKLSI | --EEHHHHHHHHHHHHHHHHHH H--- | 26 | 3 |  |  |  |
|  |  |  | DITPLIDTKAADFVEGLVQD | ----------HHHHHHHH-- | 20 | 2 |  |  |  |
| TilLV  (27) | *Tis 1* (4) | Hypothetical protein | LRIVDSDLYSERSCCWVIEKEGRVVCKST | --EE---------EEEEEE---EEEE--- | 29 | 3 | No data | QJD15201.1 | 28 |
|  |  |  | AIFLSHPFFRLLSSVVETHARSVLSKVSAVYTA | ---H—HHHHHHHHHHHHHHHH HHHHHHHHH-- | 33 | 3 |  |  |  |
|  |  |  | DLLFASCHNLSLKKSYISVASL | ---HH----------EEEE--- | 22 | 2 |  |  |  |

**Supplementary Table 1 (cont.)** Summary of all identified B-cell epitopes for constructing CMEV candidates.

| **Pathogens  (No. of total epitope)** | **Genes (No. of total epitope)** | **Protein name** | **Epitope sequence** | **Secondary structure** | **Length (amino acid residues)** | **No. of analyzed program** | **RPS (%)** | **Accession No.** | **Reference** |
| --- | --- | --- | --- | --- | --- | --- | --- | --- | --- |
| TiLV  (27) | *Tis 1* (4) | Hypothetical protein | SDLDPLVPHRLLVSESDVSKIRVARQAHLKSLGL | ---------EEEE------EEEE-HHHHHH---- | 34 | 3 | No data | QJD15201.1 | 28 |
|  | *Tis 2* (4) | Hypothetical protein | RSHTVKDVHRSLLTADKSLRKSFCFR | ----HHHHHHHHHH--HHHH------ | 26 | 3 | No data | AMR44594.1 | 6 |
|  |  |  | LDKDLPLLPIRPKLESRVAVKK | ---------------------- | 22 | 3 |  |  |  |
|  |  |  | TIEGLVVNLDDVVRGFYLDKAKVTVLSRS | --EEEEEE---HHHEEE-----EEEEE-- | 29 | 2 |  |  |  |
|  |  |  | KAMLLISCSPGTYAKKRKVAVQE | --EEEEE----------EEEE-- | 23 | 2 |  |  |  |
|  | *Tis 3* (5) | Hypothetical protein | PKATVRCILNNDATYVCSEQEY | ---EEEEEE-----EEE----- | 22 | 3 | No data | QJD15138.1 |  |
|  |  |  | PKATVRCILNNDATYVCSEQEY | ---EEEEEE-----EEE----- | 24 | 3 |  |  |  |
|  |  |  | RYTVASHKPATQKILPLPASAPLARELLMLI | --EE-----------------HHHHHHHH-- | 31 | 2 |  |  |  |
|  |  |  | RWDVILLLECLDSTRVSQAVAQH | --HHHHHHH--------HHHH-- | 23 | 3 |  |  |  |
|  |  |  | NRHRLALSVCKDEFRKGYQLAS | ---EEEEEE--HHHH------- | 22 | 2 |  |  |  |
|  | *Tis 4* (5) | Hypothetical protein | VGRSLILTSRWTEYCATCVPAL | ---EEEEE-----HHH------ | 22 | 2 | No data | QAB07938.1 | 6 |
|  |  |  | HNSLLRVCVRIEVWKARYVSLVALD | ----EEEEEEEE----EEEEEEE-- | 25 | 3 |  |  |  |
|  |  |  | QWFPYLSGDSYRACPGLVGGY | --------------------- | 21 | 2 |  |  |  |
|  |  |  | IIPPPRFLIIGHRLQIGDQVTLREL | ------EEEEEEE------EEE--- | 25 | 3 |  |  |  |
|  |  |  | DLLEECIAIQKQDGVIKCKRSGKSLYHCLKET | --HHHHHHHH----EEEE------HHHHHH-- | 32 | 3 |  |  |  |
|  | *Tis 5* (2) | Hypothetical protein | ECRDLEVYSTVLPGQCDCTR | -----EEEEEE--------- | 20 | 3 | No data | QAB07939.1 | 29 |
|  |  |  | KQVPFYGSIKVLVFRRLRVVCFKTFFY | --------HHHHHHHHHHEEEE----- | 27 | 3 |  |  |  |
|  | *Tis 6* (4) | Hypothetical protein | HFYLQDCPMSWLRVIRTLTLFSTLFS | ---------HHHHHHHHHHHHHHH-- | 26 | 3 | No data | AWK60418.1 |  |
|  |  |  | LFETSLCLSKRRPIFSTCML | -------------------- | 20 | 2 |  |  |  |
|  |  |  | TKQCWVLVESLEENHSPYKCHFSAVEVLLPA | ---EEEEEEH----------EEEEHHHH--- | 31 | 2 |  |  |  |
|  |  |  | LNPVIDLSISPPVTVRSCCKI | --------------------- | 21 | 3 |  |  |  |
|  | *Tis 7* (2) | Hypothetical protein | VPLKWKYEVSPGLPVRRVLAQ | -----EEE------HHHHH-- | 21 | 3 | No data | QMT29729.1 |  |
|  |  |  | RSALVTSTRPLTELSRVATYLEA | ---EE-------HHHHHHHHH-- | 22 | 2 |  |  |  |
|  | *Tis 10* (1) | Hypothetical protein | DEVDLEMDDCDSAIPEWARVDFN | ----------------------- | 23 | 2 | 44.5-61.1 | QIH29432.1 | 30 |
| *A. hydrophila*  (20) | *OmpA (10)* | Porin OmpA | SVATLELVMHPNRSTYVAPVAAPAP | --EEEEEEE-------EE------- | 25 | 2 | 75.5-82.3 | AVP83691.1 | 31 |
|  |  |  | EPVPEPVVVDKNFALSSDVLFAF | ------EEE----------EE-- | 23 | 3 |  |  |  |
|  |  |  | PEGVAALNTLYQQIVDVQPKDGSAVVVGYT | --HHHHHHHHHHHHHH------EEEEEE-- | 30 | 3 |  |  |  |
|  |  |  | RARTVADFLVGKGLPAGKVAI | ---HHHHHHHH---------- | 21 | 2 |  |  |  |
|  |  |  | KAKAQLIACLAPDRRVEVRVTGVQQV | -----EEEE-----EEEEEEEE---- | 26 | 2 |  |  |  |
|  |  |  | AQLAQLTMKIGLPVSESLDLY | -HHHHHHHH------------ | 21 | 3 |  |  |  |
|  |  |  | ARTVADYLVGKGLPAGKVNV | --HHHHHHH----------- | 20 | 2 |  |  |  |

**Supplementary Table 1 (cont.)** Summary of all identified B-cell epitopes for constructing CMEV candidates.

| **Pathogens  (No. of total epitope)** | **Genes (No. of total epitope)** | **Protein name** | **Epitope sequence** | **Secondary structure** | **Length (amino acid residues)** | **No. of analyzed program** | **RPS (%)** | **Accession No.** | **Reference** |
| --- | --- | --- | --- | --- | --- | --- | --- | --- | --- |
| *A. hydrophila*  (20) | *OmpA* (10) | Porin OmpA | KKELIVCLAPDRRVEVKVEGIS | ---EEEEE-----EEEEEE--- | 22 | 2 | 75.5-82.3 | AVP86728.1 | 31 |
|  |  |  | TLKPAASQALDNLFSQIVAA | ----HHHHHHHHHHHHHH-- | 20 | 2 |  | ANR98238.1 |  |
|  |  |  | SKPALIACLAPDRRVEVRLEGVS | ----EEEEE----EEEEEE---- | 23 | 2 |  |  |  |
|  | *Tdr* (6) | TonB-dependent receptor | RAPTAPATPHNVQKLDRLSLGL | -----------HHHHHH----- | 22 | 2 | 95.6 | AJQ54916.1 | 32 |
|  |  |  | DASQSALYLQDEFSLGDLALTLS | -----EEEE--------EEEE-- | 23 | 2 |  |  |  |
|  |  |  | KIKVTGSRISRVDVEGATPVVAISK | -EEEE---EEEEE------EEEE-- | 25 | 2 |  | ORJ65912.1 |  |
|  |  |  | QATVSLRGLGSERTLVLLNGK | --EEEEE-----EEEEEE--- | 21 | 3 |  |  |  |
|  |  |  | TVYVNGRYHINDSVDFVPQLIGSRVT | -EEE--EEE-----HHHHHHHH---- | 26 | 3 |  |  |  |
|  |  |  | DLNLSLDYYYIKIDDVIQLA | ----EEEEEEEEE--EE--- | 20 | 3 |  |  |  |
|  | *Aer* (4) | Aerolysin | EPVYPDQLRLFSLGQEVCGDK | --------EEEE--------- | 21 | 3 | 55.0-100.0 | KTA90213.1 / AKJ36485.1 | 33 |
|  |  |  | LSQSVRPTVPAHSKIPVKIELYK or LSQSVRPTVPARSKIPVKIELYK | ---------------EEEEEE-- | 23 | 3 |  |  |  |
|  |  |  | KFQWPLVGETELAIEIAASQS | ----------EEEEEEE---- | 21 | 2 |  | KRW48464.1 |  |
|  |  |  | TVSVEARPTVPPHSSLPVRVALYKSN | -EEEE------------EEEEEE--- | 26 | 3 |  |  |  |
| *S. agalactiae*  (14) | *Bac* (10) | C protein beta antigen | ASELVKDDSVKTTEVAAKPYPS | ------------EEE------- | 22 | 3 | 57.1-76.2 | BAE45252 | 13 |
|  |  |  | DALLELENQFNETNRLLHIKQ | --HHHHHHHHHHHHHHEE--- | 21 | 2 |  |  |  |
|  |  |  | PELKQLEEEAHSKLKQVVED | ---HHHHHHHHHHHHHHH-- | 20 | 2 |  |  |  |
|  |  |  | EKYFLTPFNKIKQIVDDLDK | -------HHHHHHHHH---- | 20 | 2 |  |  |  |
|  |  |  | KIAVSKYMSKVLDGVHQHLQK | --HHHHHHHHHHHHHHHHH-- | 21 | 3 |  |  |  |
|  |  |  | APRVPESPKTPEAPHVPESP | -------------------- | 20 | 2 |  |  |  |
|  |  |  | TPDVPKLPDVPKLPDVPKLPDAPKLP | -------------------------- | 26 | 3 |  |  |  |
|  |  |  | NTKVTVVFDKPTDADKLHLKEVT | --EEEEEE--------EEE---- | 23 | 3 |  |  |  |
|  |  |  | RTVRLALGQTGSDVHVYHVKE | --EEEEE------EEEEEE-- | 21 | 3 |  |  |  |
|  |  |  | VENGQVVFKTNHFSLFAIKTL | ----EEEEE-----EEEE--- | 21 | 3 |  |  |  |
|  | *Rib* (1) | Surface protein Rib | AKVVVTYPDGSKDTVYVTVKVVDP | -EEEEE-------EEEEEEEE--- | 24 | 3 | 57.1-76.2 | EAO72273 |  |
|  | *Sip* (1) | Group B Streptococcal surface immunogenic protein | EVKSVPVAQKAPTATPVAQP | ---EE--------------- | 20 | 3 | 57.1-76.2 | AUP09114 |  |
|  | *Csf* (1) | Cell surface protein | VQGVYLLKTPLPLPEYYIGLNVYF | ---EEEE--------EEEE--E-- | 24 | 3 | 57.1-76.2 | AIK73093 |  |
|  | *Spb* (1) | Surface protein Spb1 | GTEKVYQYVIKDTMPSASVVDL | ---HHHHHHHH----------- | 22 | 3 | 57.1-76.2 | WP_000913277 |  |

Note: "H” and “E” in secondary structure indicate α-helix and β-sheet structures; The number in blanket indicates total number of identified epitopes of each pathogen

**Supplementary Table 2** The supported data for the selected B-cell epitopes of constructed CMEV candidates.

| **Pathogens** | **Epitope sequence** | **Secondary structure** | **Hydropho-bicity (%)** | **Length (amino acid residues)** | **Position from N-terminus** | **No. of analyzed program** | **RPS (%)** | **Reference** |
| --- | --- | --- | --- | --- | --- | --- | --- | --- |
| *E. tarda* | AQALPSLAPMLEKVMPSVVSINVE | ------HHHHHHHHHHHHEEEE-- | 66.67 | 24 | 35-58 | 3 | 89.0 | 16 |
|  | EKIAVVNVASVFQQLPQRDAVAKQ | --EEEEEHHHHHHH---------- | 54.17 | 24 | 37-60 | 3 | 83.0 | 17 |
| *F. columnare* | RQLYTSQQAYLQSMVEVITK | ---HHHHHHHHHHHHHHH-- | 35.00 | 20 | 410-429 | 3 | 68.0 | 21 |
|  | KFEQLADSLVKRSMEPVVKAL | --HHHHHHHHHHHHHHHHH-- | 52.38 | 21 | 298-318 | 2 | Increase survival rate > 30% | 22 |
|  | DIDEVILVGGSTRIPVIQEQVEKF | ---EEEEE------HHHHHHHH-- | 45.83 | 24 | 327-350 | 3 |  |  |
| *F. noatunensis* | RKPYSVILLDEVEKAHADIFNILLQVLDD | ----EEEEE--HHH-HHHHHHHHHHHH-- | 51.72 | 29 | 670-698 | 3 | No data | 23 |
|  | EIAKIQIKRLEKRLADLSIGLEV | --HHHHHHHHHHHHHH------- | 47.83 | 23 | 774-796 | 2 |  |  |
|  | KYIQKVITVIDKLIDLQVNSII | -HHHHHHHHHHHHHHH------ | 50.00 | 22 | 71-92 | 3 |  |  |
|  | HGIALLRKAIEAPLRQIVSNAGGESSVVVNQVK | --HHHHHHHHHHHHHHHHHH----HHHHHH--- | 48.48 | 33 | 438-470 | 3 | 22.0-50.0 | 24 |
| *S. iniae* | DYDYVIKHKEDIVKHYSDIFVDLQS | ---EEHH-HHHHHHHHHHHEEE--- | 32.00 | 25 | 319-343 | 3 | 69.7 | 27 |
|  | KYNQLLRIEDQLGEVAQYKGIK | ---HHHHHHHHHHHH------- | 31.82 | 22 | 407-428 | 2 | 63.0 | 6 |
|  | KRVLVMDKVADQLIANVKTLVDKLSI | --EEHHHHHHHHHHHHHHHHHHH--- | 53.85 | 26 | 288-313 | 3 | 63.0-77.8 | 26 |
| TiLV | AIFLSHPFFRLLSSVVETHARSVLSKVSAVYTA | ---H—HHHHHHHHHHHHHHHHH HHHHHHHH-- | 54.55 | 33 | 153-188 | 3 | No data | 28 |
|  | KQVPFYGSIKVLVFRRLRVVCFKTFFY | --------HHHHHHHHHHEEEE----- | 51.85 | 27 | 295-321 | 3 | No data | 29 |
|  | HFYLQDCPMSWLRVIRTLTLFSTLFS | ---------HHHHHHHHHHHHHHH-- | 50.00 | 26 | 2-27 | 3 |  |  |
| *A. hydrophila* | PEGVAALNTLYQQIVDVQPKDGSAVVVGYT | --HHHHHHHHHHHHHH-----EEEEEE-- | 46.67 | 30 | 223-252 | 3 | 75.5-82.3 | 30 |
|  | TVYVNGRYHINDSVDFVPQLIGSRVT | -EEE--EEE-----HHHHHHHH---- | 38.46 | 26 | 293-320 | 3 | 95.6 | 32 |
|  | TVSVEARPTVPPHSSLPVRVALYKSN | -EEEE------------EEEEEE--- | 50.00 | 26 | 299-324 | 3 | 55.0-100.0 | 33 |
| *S. agalactiae* | DALLELENQFNETNRLLHIKQ | --HHHHHHHHHHHHHHEE--- | 38.10 | 21 | 146-166 | 2 | 57.1-76.2 | 13 |
|  | KIAVSKYMSKVLDGVHQHLQK | --HHHHHHHHHHHHHHHHH-- | 38.10 | 21 | 747-767 | 3 |  |  |
|  | GTEKVYQYVIKDTMPSASVVDL | ---HHHHHHHH----------- | 40.91 | 22 | 214-235 | 3 |  |  |

Note: "H” and “E” in secondary structure indicate α-helix and β-sheet structures

**Supplementary Figure 1.** Amino acid type prediction of the CMEVs. [A-C] The amino acid profile of OSLM0467, OSLM2629, and OSLM4294. Orange, green, red, and blue represented amino acid types of small nonpolar, hydrophobic, polar, and aromatics plus cysteine, respectively.


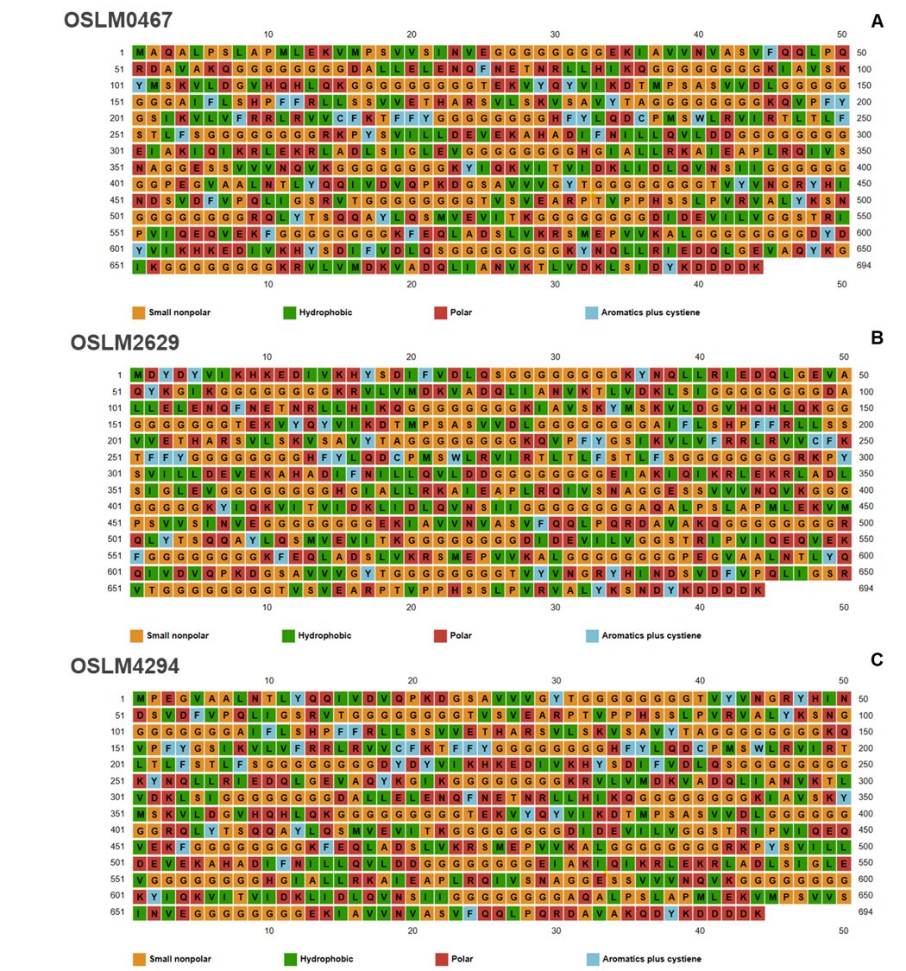


**Supplementary Figure 2.** *In silico* simulation of the immune responses after vaccination with the selected CMEVs. [A-C] The district isotypes of the PLB cell population (IgM, IgG1, IgG2), [D-F] B-cell population per state, [G-I] T-Helper cell induction, [K-M] Cytotoxic T-cell proliferation, and [N-P] NK cell stimulation after post-vaccination with OSLM0467, OSLM2629 and OSLM4294.


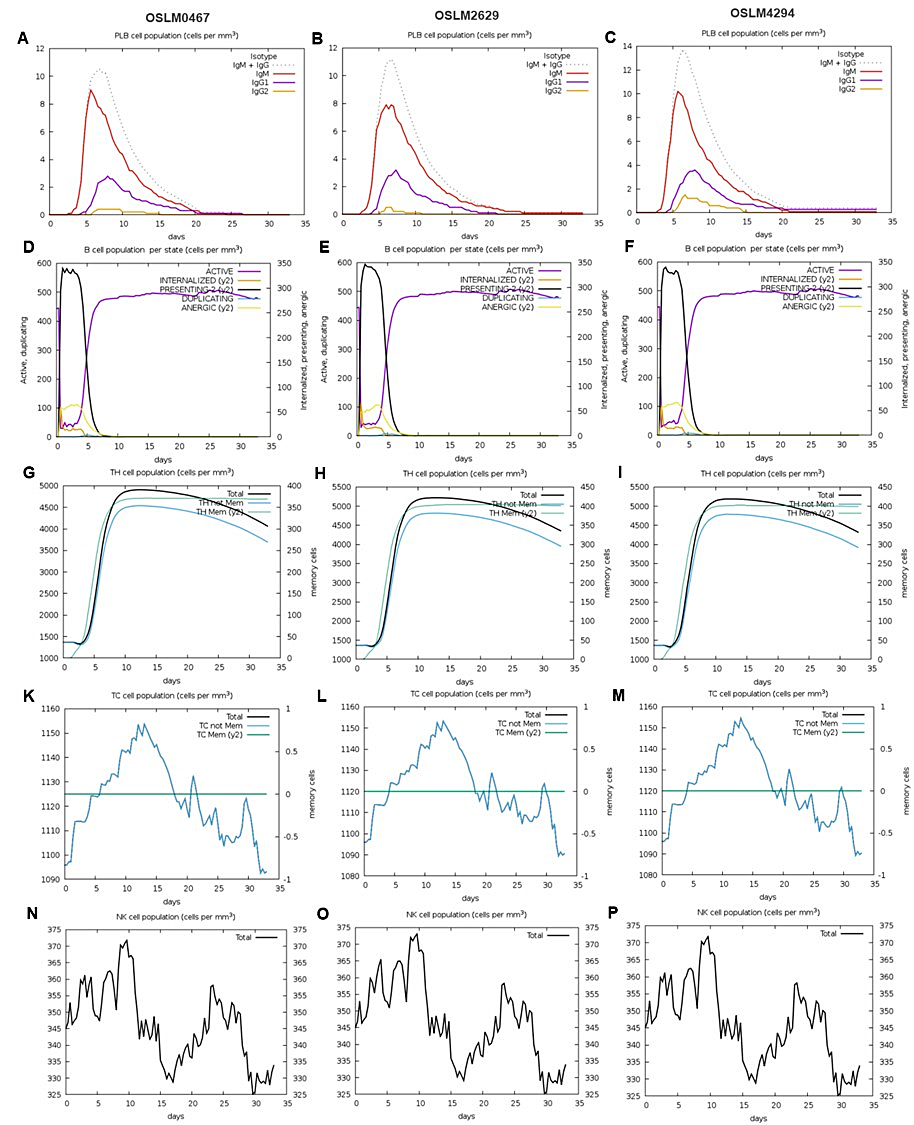


**Supplementary Figure 3.** The RMSF score (Å) of the CMEV-TLR4 complexes was evaluated through the CABS-flex 2.0 server. The A- and B-chains indicated amino acid residues of the TLR4 receptor, while the C-chain indicated the CMEV molecule.

**
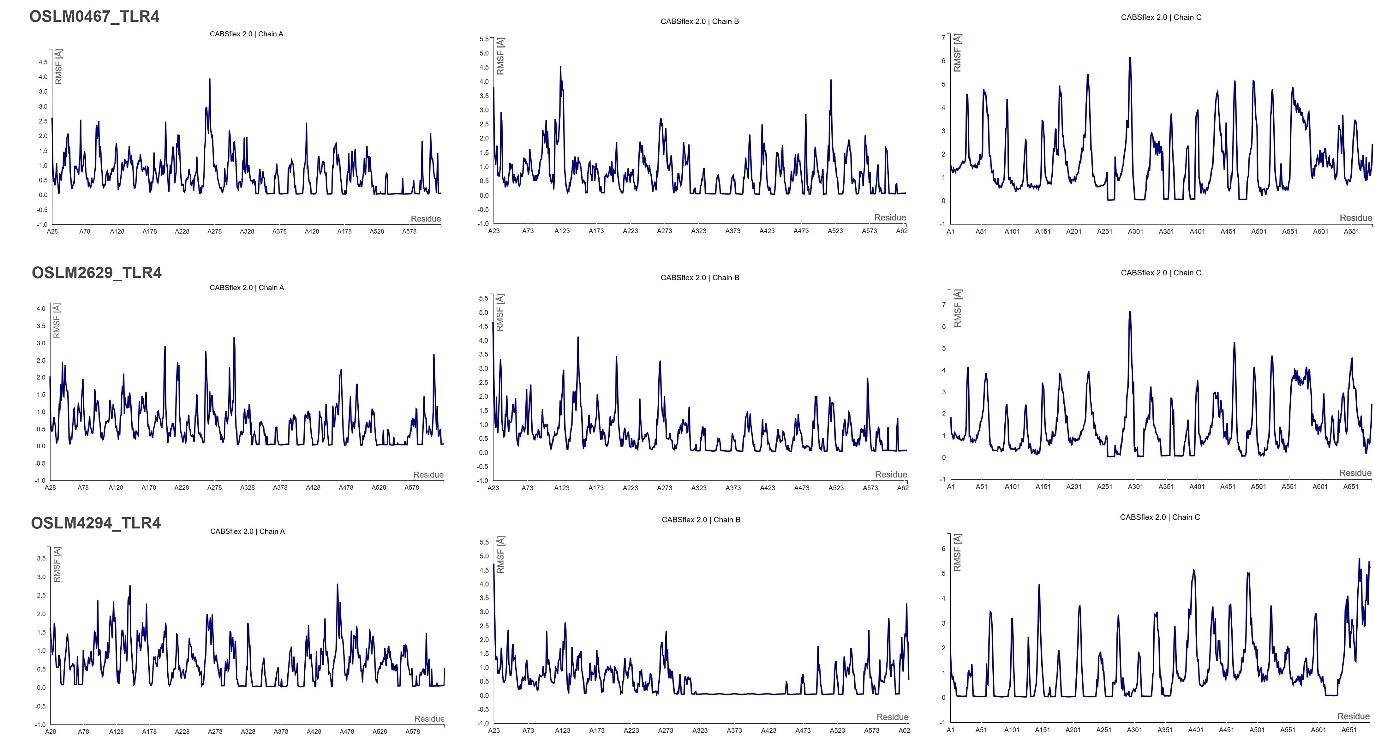
**

**Supplementary Figure 4.** Histogram of the codon-optimized CMEV candidates based on codon bias of *E. coli* and Nile tilapia (*Oreochromis niloticus* *113 CDS* gene). [A-C] The codon quality distribution of the OSLM0467, OSLM2629, and OSLM4294 which adjusted to *E. coli* codon usage, and [G-I] adapted to *O. niloticus* codon bias. The plot explained the percentage of sequence codons that fall into a certain quality class. The CAI of the optimized CMEV genes was 0.94 and 0.87 for *E. coli* [A-C] and *O. niloticus* [G-I] adaptations. The average GC content in the ideal range (between 30%-80%) of CMEVs adjusted to *E. coli* was observed in [D-F] and adapted to *O. niloticus* was noticed in [J-L].


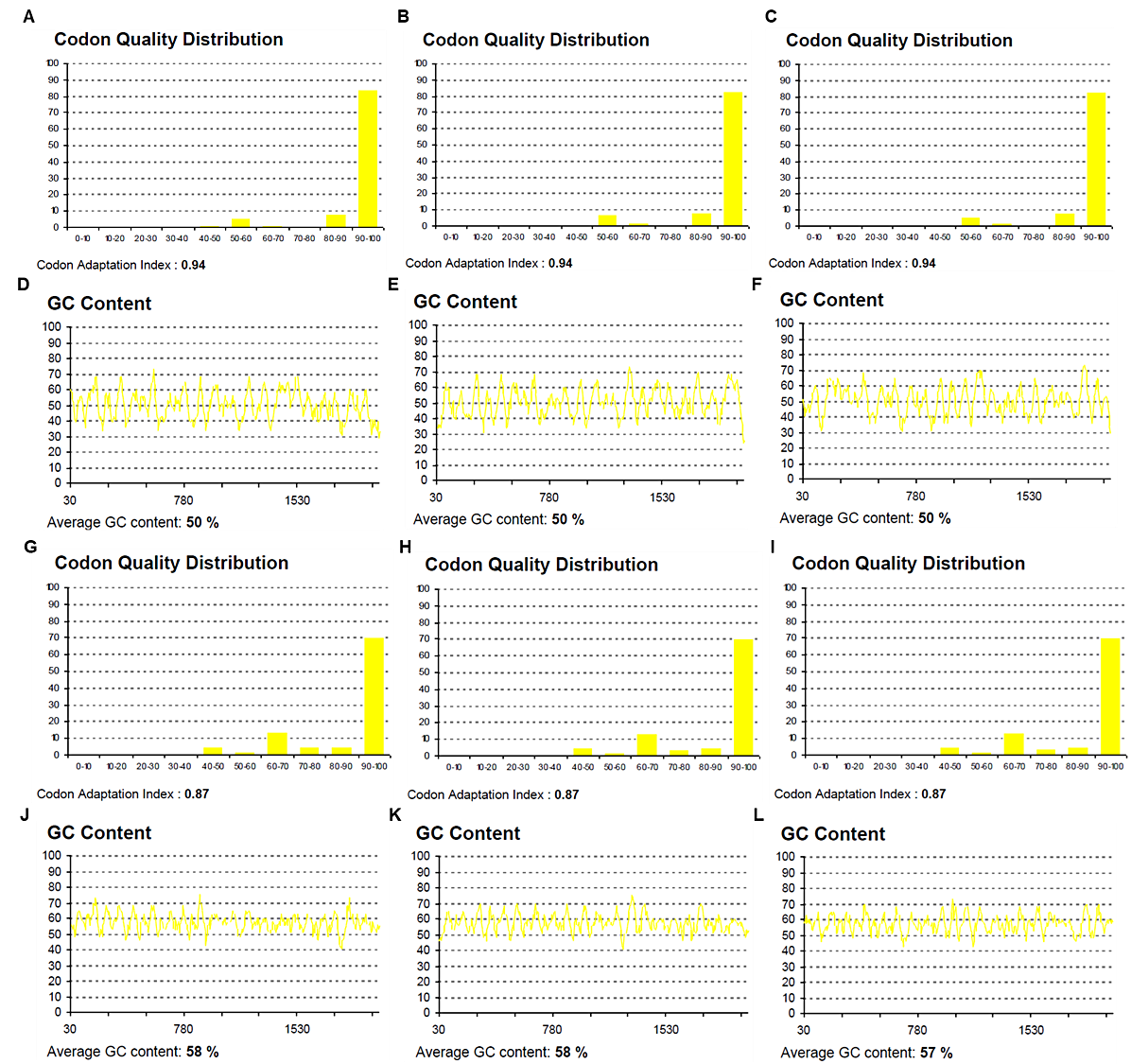


**Supplementary Figure 5.** The MFE secondary structure of the optimized CMEVs’ RNA folding. [A-C] The predicted RNA structures of OSLM0467, OSLM2629, and OSLM4294, respectively. Green, red, yellow, blue, and orange colors indicated the structures of stems (canonical helices), multiloops (junctions), interior loops, hairpin loops, and 5' and 3' unpaired regions.


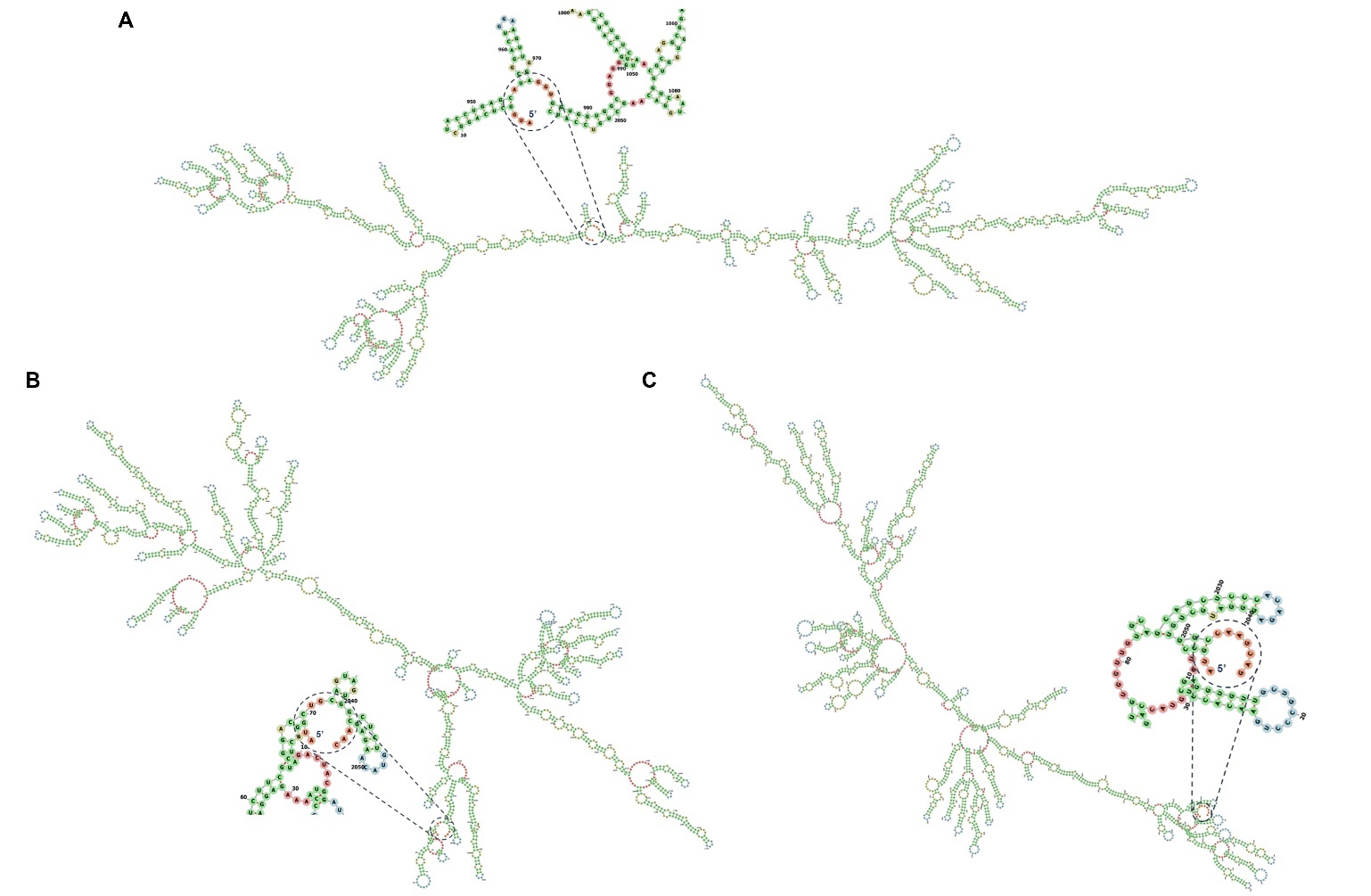

Supplement: Supplementary file 1 — Supplementary Information. [file 41598_2024_64383_MOESM1_ESM.docx]
